# Community-based interventions for enhancing access to or consumption of fruit and vegetables among five to 18-year olds: a scoping review

**DOI:** 10.1186/1471-2458-12-711

**Published:** 2012-08-30

**Authors:** Rebecca Ganann, Donna Fitzpatrick-Lewis, Donna Ciliska, Leslea Peirson

**Affiliations:** 1 Effective Public Health Practice Project, McMaster University, Hamilton, ON, Canada

## Abstract

**Background:**

Low fruit and vegetable ( FV) consumption is a key risk factor for morbidity and mortality. Consumption of FV is limited by a lack of access to FV. Enhanced understanding of interventions and their impact on both access to and consumption of FV can provide guidance to public health decision-makers. The purpose of this scoping review is to identify and map literature that has evaluated effects of community-based interventions designed to increase FV access or consumption among five to 18-year olds.

**Methods:**

The search included 21 electronic bibliographic databases, grey literature, targeted organization websites, and 15 key journals for relevant studies published up to May 2011. Retrieved citations were screened in duplicate for relevance. Data extracted from included studies covered: year, country, study design, target audience, intervention setting, intervention strategies, interventionists, and reported outcomes.

**Results:**

The search located 19,607 unique citations. Full text relevance screening was conducted on 1,908 studies. The final 289 unique studies included 30 knowledge syntheses, 27 randomized controlled trials, 55 quasi-experimental studies, 113 cluster controlled studies, 60 before-after studies, one mixed method study, and three controlled time series studies. Of these studies, 46 included access outcomes and 278 included consumption outcomes. In terms of target population, 110 studies focused on five to seven year olds, 175 targeted eight to 10 year olds, 192 targeted 11 to 14 year olds, 73 targeted 15 to 18 year olds, 55 targeted parents, and 30 targeted teachers, other service providers, or the general public. The most common intervention locations included schools, communities or community centres, and homes. Most studies implemented multi-faceted intervention strategies to increase FV access or consumption.

**Conclusions:**

While consumption measures were commonly reported, this review identified a small yet important subset of literature examining access to FV. This is a critically important issue since consumption is contingent upon access. Future research should examine the impact of interventions on direct outcome measures of FV access and a focused systematic review that examines these interventions is also needed. In addition, research on interventions in low- and middle-income countries is warranted based on a limited existing knowledge base.

## Background

Low fruit and vegetable (FV) consumption is one of the top 10 global risk factors for mortality according to the World Health Organization (WHO) [1]. Increased FV consumption can help protect overall health status and reduce both disease risk and burden [2]. Fruit and vegetable intake among children is of particular interest due to growing recognition of the importance of nutrition for growth, development, and prevention of chronic diseases such as cardiovascular disease and obesity [2]. A number of studies have shown that childhood FV consumption patterns and preferences are predictive of patterns in adolescence and adulthood [3-6]. It has been estimated that 2.7 million lives could be saved each year through increased and adequate FV consumption. In addition, this increased consumption of FV would decrease the worldwide non-communicable disease burden by almost 2% [7].

Consumption of FV is limited by a lack of access to FV, which is a conspicuous issue facing low- and middle-income countries, but also affects high-income countries [8]. While FV intake is directly associated with socioeconomic status, many individuals do not meet recommended guidelines for FV intake regardless of country of origin and income status [8]. A systematic review of potential determinants of FV intake and intervention strategies found that availability and accessibility of FV and preferences had the most consistent positive relationship with FV consumption [9]. Another more recent systematic review of determinants of fruit and vegetable consumption among children and adolescents identified many individual level determinants including direct gradients associated with intake and socioeconomic status, home availability and accessibility, and parental intake patterns [8]. The environmental factors that influence consumption of FV extend beyond the individual to include: physical, economic and social factors; supply, availability and accessibility (includes costing); availability of FV in stores in the local community, schools, community-based programs; and policies at global, regional, national and local levels [8-11]. Nutrition knowledge, preferences, and self-efficacy are also associated with increased intake of FV [8,12,13] and therefore have been included as secondary outcomes of interest for this review. Enhanced understanding of relevant intervention research and its impact on both access to and consumption of FV, as well as chronic disease health indicators, can provide guidance to public health decision-makers and policy-makers in the establishment and maintenance of effective and supportive nutritional programs.

Three reviews have previously examined the effect of community interventions to increase FV consumption, however, one review has not been updated in over 10 years [14] and the two others were limited to school-based interventions in high-income countries [15,16]; in these the previous reviews, the important issue of access to FV was not addressed [14-16]. The purpose of this scoping review is to identify and map literature that has evaluated the effects of community-based interventions designed to increase FV access and/or consumption among five to 18-year olds. The effectiveness of upstream interventions targeting children and adolescents (18 years and under) is of particular interest to decision makers since FV consumption patterns established in childhood tend to persist through adulthood [3-6]. We are aware of a potentially complementary review that intends to examine interventions to increase FV consumption in preschool children (under 5 years) [17] and therefore focused on 5 to 18 year olds. Due to the large scope of this review topic, interventions focused solely on adults or specialized populations such as those with a specific chronic disease were considered beyond the scope of this review.

The initial step in this scoping review involved defining the research question in a PICOS (Population-Intervention-Comparison-Outcome-Study Design) format. The PICOS question was defined by the research team and refined in collaboration with two public health librarians who subsequently implemented the search.

## Methods

### Inclusion criteria

#### Participants

This review includes populations from low-, middle-, and high-income countries and focuses on children aged five to 18 years.

##### Interventions

We included interventions delivered to anyone that brings about changes in FV access and consumption for five to 18 year olds (i.e., parents, communities, and others within the population, including children themselves). The following types of community-based interventions were included:

•  Nutrition-friendly schools initiatives

•  Child nutrition programs such as breakfast/lunch and summer food service programs

•  Community programs (e.g., community gardens)

•  Health education related to increased FV consumption

•  Economic supplements and subsidies to purchase FV, including subsidies for schools and food stamp programs

•  Environmental school change strategies (e.g., changing the types of foods provided in cafeterias or vending machines)

•  Environmental interventions/industry partnerships focused on point-of-purchase (e.g., restaurants, grocery store distributors and retailers); this might include campaigns to draw attention to healthier products in grocery stores or to highlight the benefits of certain foods or within store promotions and costs

•  Population level initiatives (e.g., agricultural policies)

•  Internet, telephone and media interventions

•  Farm-to-school programs that use locally produced foods

•  Social marketing campaigns

•  Policies that affect accessibility factors

•  Policies that seek to increase FV consumption (i.e., school board level, provincial/national level).

#### Locations

Intervention locations included: homes, schools, health departments, religious institutions, family/child centres, community/recreation centres, non-governmental organizations, and primary healthcare settings. We excluded programs or strategies delivered through hospitals; outpatient clinics located within hospital settings; commercial programs, such as *Health Check*; universities/colleges; and metabolic or weight loss clinics.

#### Outcomes of interest

Our primary outcomes included measures of both access to and consumption of fruit, vegetables, or both. Evidence of intervention effects included: measures at individual, family, school or community levels. Measures of FV access included: FV supply (i.e., market inventory); and change in food environments, food disappearance, and food sales (in cafeterias and grocery stores). Food supply measures included information about which food items are distributed to different regions and areas. Market inventory refers to records a food supply organization keeps about which foods are being ordered or are available. Measures of FV consumption included: diet and food intake records, self-reported and/or reported by parents, teachers or both; food frequency questionnaires/balance sheets; food wastage and plate waste; and micronutrient measures (i.e., biomarkers of exposure to FV).

Our secondary outcomes included: awareness of importance/impact of FV consumption among targeted individuals, attitudes towards consumption of FV, general health measures including changes in weight, and adverse outcomes or unintended consequences.

#### Study designs

Acceptable designs for this review included systematic reviews (included research syntheses and meta-analyses), randomized and non-randomized studies (including cluster-controlled and controlled time series), interrupted time series (to assess changes that occur over time), and before-after studies with controls. Relevant clusters within studies, included school units, classrooms or communities rather than individuals as the unit of analysis.

### Search strategy

Our search strategy included: electronic bibliographic databases; grey literature databases; reference lists of key articles; targeted internet searching of key organization websites; and hand searching of key journals.

We searched the following databases, adapting search terms according to the requirements of individual databases in terms of subject heading terminology and syntax: MEDLINE and Pre-MEDLINE; EMBASE; CINAHL and Pre-CINAHL; the Cochrane Central Register of Controlled Trials (CENTRAL); the Cochrane Public Health Group Specialized Register; PsycINFO; Dissertation Abstracts; ERIC; Effective Public Health Practice Project Database; Sociological Abstracts; Applied Social Sciences Index; CSA Worldwide Political Science Abstracts; ProQuest (ABI/Inform Global); PAHO Institutional Memory Database; WHO Database on Child Growth and Malnutrition; Healthstar; Current Contents; ScienceDirect; and LILACS. The original search was conducted on August 17, 2010 and was updated on May 31, 2011, searching each database from its beginning. Our search strategy for the electronic databases is shown in Appendix 1 (Additional file 1).

We used the Grey Matters search tool, Federated Search for applicable policy documents, the System for Grey Literature in Europe and the Global Health Database to search for relevant grey literature. We conducted a hand search of the reference lists of all relevant articles for any additional references. We also searched key sites, including the World Health Organization ( http://www.who.int/en/), the Food and Agriculture Organization of the United Nations ( http://www.fao.org/), and Pan American Health Organization ( http://new.paho.org/). Further, we had searched the following journals (for the 12-month period prior to the date of search [Aug. 17, 2010]): *Health Policy*; *Journal of Public Health Policy*; *Journal of Health Politics, Policy, and Law*; *Health Economics, Policy, and Law*; *American Journal of Clinical Nutrition*; *Journal of Health Services Research*; *American Journal of Public Health; Journal of the American Dietetic Association*; *Nutrition Reviews*; *Maternal and Child Nutrition*; *Nutrition and Dietetics*; *Nutrition Research*; *Public Health Nutrition*; *American Journal of Preventive Medicine* and *Journal of Hunger and Environmental Nutrition.* Journal selection for hand searching was guided by consultation with experts and our review advisory committee.

### Study selection

A librarian conducted a search for relevant literature. The search strategy identified titles and abstracts. Teams of two reviewers conducted relevance screening to eliminate obviously irrelevant studies; each person independently reviewed titles and abstracts for relevance screening. All articles selected by either team member were retrieved for full text review. For citations with no abstract, the full article was retrieved for full text relevance screening.

Review teams independently examined the full text of retrieved articles for relevance. A third reviewer was consulted to resolve any disagreements related to inclusion of articles. Studies excluded following full text reviews and reasons for exclusion were documented. Articles in English, French and Spanish were reviewed at the inclusion screening stages (title/abstract and full text review).

### Data extraction and sorting

For all included studies, data were extracted by two reviewers and included: year of publication, study design, types of outcomes reported and research location. When there was more than one publication per study, these citations were grouped into ‘projects’. Only articles published in English and French underwent data extraction due to the fluency of available reviewers.

## Results

### Citation retrieval

**Figure 1:**
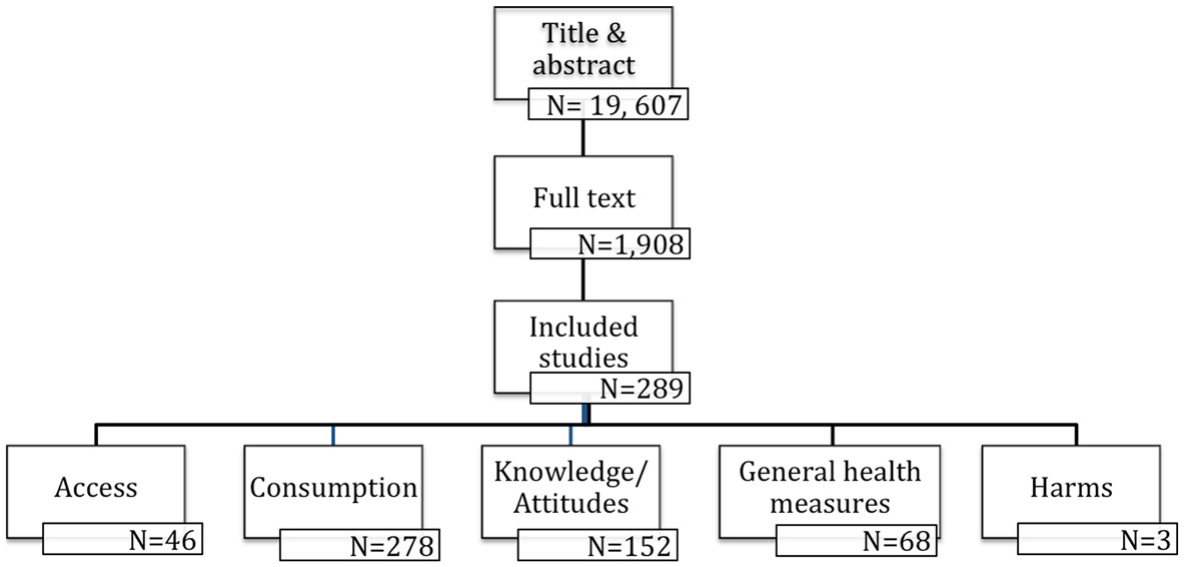
Flowchart of screening process for unique citations retrieved, number of included studies at each level of screening, and outcomes reported in included studies.

The search strategy retrieved nearly 23,000 citations, which were reviewed by research assistants to remove duplications. Of the citations identified, 22,287 (97.3%) were found through published literature databases, 156 (0.7%) through grey literature searching, and 468 (2.0%) through hand searching relevant journals. Two reviewers independently examined the titles and abstracts of 19,607 unique citations for relevance. Following title and abstract review, 17,699 (90.3%) citations were excluded and 1,908 (9.7%) remained to undergo full text relevance screening. Following full text review, 1,619 (84.9%) studies were excluded with 289 (15.1%) unique studies remaining. Of the citations excluded during full text review, 52 (3.2%) were excluded because they were published in a language other than English or French, 366 (22.6%) had target audiences that did not include children aged five to 18 years or persons who had influence over FV access or consumption for children, 232 (14.3%) did not use a study design appropriate for evaluating interventions, 638 (39.4%) did not evaluate a relevant intervention or policy, 236 (14.6%) did not have baseline comparison data, and 95 (5.9%) did not report outcomes of interest for five to 18 year olds. See Figure 1 for a flowchart of literature retrieved, levels of screening, included studies, and types of outcomes.

The final 289 unique studies were found in the form of journal articles and reports. The published citations appeared in 100 periodicals with 89 published in five journals. These included 12 articles published in the *Journal of Nutrition Education & Behavior,* 14 in the *Journal of Nutrition Education*, 17 in the *Journal of the American Dietetic Association,* 19 in *Preventive Medicine,* and 27 in *Public Health Nutrition.* The year of publication for included articles ranged from 1970 to 2011, with 230 studies published during or since 2001. See Appendix 2 (Additional file 2) for details of all included studies, such as author(s), title, year, location, design, target population, and types of outcomes measured. As seen in Appendix 2, very few studies from low- or middle-income countries were identified.

### Study designs and outcomes

The final 289 unique studies included 30 knowledge syntheses (including narrative systematic reviews and meta-analyses) [9,14,15,18-44], 27 randomized controlled trials [45-71], 55 quasi-experimental studies [72-126], 113 cluster controlled studies [94,127-237], 60 before-after studies [238-306], one mixed method study [307], and three controlled time series studies [308-310]. Several of studies had multiple publications reporting results (e.g., outcomes reported at different time points): Ammerman et al. [18,19,311], Bere [139,312,313]; Bere et al. [140,314,315], Byrd-Bredbenner et al. [146,316], Ciliska et al. [15,317], Chen et al. [48,318], Colby [247,319], Covelli [79,320], Cullen et al. [253,321], Gortmaker et al. [163,322], Haarens et al. [166,323], Hendy et al. [55,324], Hollar et al. [96,325,326], Hopper et al. [174,327], Jimenez et al. [100,328,329], Latimer [273,330], Lautenschlager and Smith [275,331], Lytle et al. [191,332,333], McCormick et al. [285,334], Nicklas et al. [198,335], Parmer et al. [203,336], Reinaerts et al. [217,337], Tak et al. [121,338,339], Tanner et al. [120,340], Taylor et al. [229,341], Thomas et al. [42,342], Thompson et al. [231,232,343], Walker [267,303], Wardle et al. [69,344], and Wrigley [306,345]. The number of citations for each study design and the reported outcome measures are summarized in Table 1. All study tallies included in the following sections and associated (Tables 2, 3, 4 and 5) include both knowledge syntheses and primary studies.

**Table 1:** Study design and outcomes

**Study design**	**Access**	**Consumption**	**Knowledge/ Attitudes/ Awareness**	**General health measures**	**Harms**	**Total Studies by Design**
**Systematic Review**	8	30	18	9	0	30
**RCT**	2	27	14	12	0	27
**Quasi-Experimental**	6	51	26	12	0	55
**Cluster Controlled**	11	107	59	25	3	113
**Before-After (no control)**	18	59	31	9	0	60
**Mixed Method**	1	1	1	0	0	1
**Controlled Time Series**	0	3	3	1	0	3

**Table 2:** Target audience and outcomes

**Audience**	**Access**	**Consumption**	**Knowledge/ Attitudes/ Awareness**	**General health measures**	**Harms**
**5 - 7 year olds**	16	109	55	21	1
**8 - 10 year olds**	30	175	98	40	3
**11 - 14 year olds**	29	187	102	44	3
**15 - 18 year olds**	12	71	42	21	0
**Parents**	15	57	33	22	1
**Teachers/ school personnel**	3	10	9	6	1
**Other service providers**	3	3	2	1	0
**General public**	6	14	4	2	0

**Table 3:** Intervention locations and outcomes

**Location**	**Access**	**Consumption**	**Knowledge/ Attitudes/ Awareness**	**General health measures**	**Harms**
**School**	33	229	130	47	3
**Supermarket**	2	9	6	2	0
**Religious institution**	0	2	2	1	0
**Community**	12	37	22	15	1
**Camps**	2	5	5	2	0
**Primary care setting**	0	5	1	2	0
**Home**	9	38	22	10	1
**Internet**	2	5	4	3	0
**Other**	5	26	15	9	0

**Table 4:** Intervention strategies and outcomes

**Intervention Strategy**	**Access**	**Consumption**	**Knowledge/ Attitudes/ Awareness**	**General health measures**	**Harms**
**Class series**	14	172	107	35	2
**Community-wide intervention**	9	31	16	14	0
**Comprehensive school health**	4	17	16	6	0
**Group discussion**	6	43	29	13	0
**Individual counseling/ teaching**	3	32	16	9	0
**Interactive approach**	25	166	100	52	1
**Parent involvement**	16	105	63	34	1
**Pedagogical/ lecture approach**	2	28	14	10	0
**Peer-led**	2	19	16	5	0
**Community garden**	4	19	13	2	0
**Policy**	16	37	15	8	1
**Marketing**	14	51	31	15	1
**Educational written material**	19	127	76	36	2
**Behavior modification**	8	67	38	23	1
**Creating supportive environments**	15	79	49	27	0
**Provision of fruit and/or vegetables**	23	116	64	20	2
**Other**	32	144	76	32	2

**Table 5:** Intervention delivery and outcomes

**Intervention delivered by**	**Access**	**Consumption**	**Knowledge/ Attitudes/ Awareness**	**General health measures**	**Harms**
**Teacher**	14	130	85	31	3
**Principal/school administration**	9	30	14	6	1
**Community lay person**	3	15	11	4	0
**Peer**	1	17	15	4	0
**Farmer**	0	2	2	0	0
**Dietician**	2	18	8	7	0
**Other health professional**	2	23	10	8	0
**Other school personnel**	10	49	29	9	2
**Health department or Ministry of Health**	3	11	3	2	0
**Other**	29	148	80	37	1

### Intervention target populations and outcomes

The target audiences for interventions may have included sub-groups within the age range of five to 18 years; however, adults who influence children’s nutritional access or consumption may also have been targeted. For interventions that targeted these ‘other’ audiences, the articles needed to report outcomes for five to 18 year olds to be included in this review. Of the unique studies, 110 targeted five to seven year olds, 175 targeted eight to 10 year olds, 192 targeted 11 to 14 year olds, 73 targeted 15 to 18 year olds, 55 targeted parents, 12 targeted teachers, five targeted other service providers, and 13 targeted the general public. For a breakdown of outcomes reported in both knowledge syntheses and primary studies by target audience see Table 2. Additional file 2 summarizes target audiences for each individual study together with other study features.

### Intervention locations and outcomes

Each unique citation was also examined to identify the locations in which interventions were delivered. Interventions were delivered in a wide variety of locations: schools (n = 233), supermarkets (n = 9), religious institutions (n = 2), community or community centres (n = 37), camps (n = 7), primary care settings (n = 5), homes (n = 38), by internet (n = 6), and other locations (n = 26). The other locations included after school programs, Boy and Girl Scout troop meetings, child care centres, farms or farmers’ markets, pediatricians’ offices, YMCAs and youth programs. The outcomes measured in various locations of intervention delivery are shown in Table 3 (includes knowledge syntheses and primary studies). Many studies were delivered in multiple locations such as in schools plus community [30,40,42,100,246,282], plus home [26,31,94,102,110,144,145,154,174,189,197,205,254,281,327,346], plus supermarkets [137], plus after school programs [43], plus the internet [103], plus other [9,27,67,195,257,286], or schools plus two or more other locations [19,23,24,29,35-38,88,157,159,161,317]. Several other studies combined a general community location plus a supermarket [82], camp [265], home [20,135], religious institution [53], other [181], or home plus internet components [231]. Three studies implemented interventions in primary care settings plus the home [25,62,63]. Others primarily targeted the home with other components delivered either by internet [251], in other locations [151,231,270], or both [46]. One study delivered an intervention within a religious institution combined and a camp [247].

### Intervention strategies and outcomes

Most studies implemented multi-faceted intervention strategies with only approximately 10% of studies implementing individual strategies to increase FV access or consumption. An example of a multiple intervention strategy is an educational series delivered primarily in a school with an added homework component to engage parents. The outcomes measured using different intervention strategies are shown in Table 4 (knowledge syntheses and primary studies).

### Intervention delivery and outcomes

Unique studies were also examined to determine who had delivered interventions. Most often teachers (n = 130), school administrators (n = 32) and/or other school personnel (n = 49) were involved in delivery. In other cases, dieticians (n = 19), health departments or health ministries (n = 11), and other health professionals (n = 23) were responsible for implementation. Community lay persons and peers were involved in delivering interventions in 12 and 15 studies, respectively. In some studies the researchers implemented the interventions (n = 13), whereas in many others it was not stated who delivered the interventions. A breakdown of the outcomes and by whom the interventions were delivered (knowledge syntheses and primary studies) is summarized in Table 5. Some studies had interventions that were delivered by multiple individuals, such as teachers plus another community or school individual (e.g., administrator, health professional, other school personnel, researcher) [21,26,29,37,38,41,52,59,72,97,103-105,118,121,131,134,137,140,144,157,161,198,200,214,217,218,226,249,258,261,274,291,301,309,310,317,338,342]; teachers plus 2 or more other individuals [15,23,28,31,36,43,74,75,88,102,110,119,127,137,159,166,172,175,199,202,205,213,219,246,262,276,299]; Health Department or Ministry of Health together with school administrators or other school personnel [224,264,307]; peers plus teachers and/or administrators [16,30,35,40,55,143,155,190]; administrators and other school personnel [193,297,305]; peers plus other school personnel [55,160]; peers plus administrators plus other school personnel [173]; peers plus dietician [187,277]; peers plus community members [124]; dietician plus other school or health professionals [19,115,250,318]; and other school or health professionals plus other community or health providers [191,230,286].

### Knowledge syntheses summary

This scoping review identified 30 systematic reviews, all of which reported on consumption outcomes; only eight reported on access outcomes as well. Approximately two-thirds reported on our secondary outcomes of interest that included knowledge, attitudes, awareness, and general health measures. None of the included systematic reviews reported on harms.

## Discussion

The Cochrane Public Health Group acknowledges that a scoping review is a critical step in defining a systematic review question [347]. We identified a large volume of interventional research found within peer-reviewed and grey literature associated with FV access and consumption. Using a scoping review process [347], we categorized these studies with respect to outcomes based on a number of parameters such as study design, target audience, intervention location, intervention strategy, and intervention deliverer.

The predominant outcome measure was consumption. In comparison, harm was included as an outcome measure in an extremely small number of studies, which may indicate that there are few risks or potential harms associated with interventions used to increase FV consumption. It also possible that harms were overlooked given that few of these studies evaluated interventions implemented in low- or middle-income countries where populations could be more vulnerable. The bias of studies toward high-income countries and not low- and middle-income countries warrants further investigation as intervention effectiveness may vary across these populations.

While consumption measures were commonly reported, this review identified a small yet important subset of literature examining the effectiveness of interventions that increase access to FV. We believe this to be a relatively overlooked but critically important issue since consumption of FV is contingent upon access to them. A number of articles discussed FV accessibility; however, many of these studies lacked before-after comparison data or other comparison groups. These studies were excluded during full text relevance screening since they did not evaluate the impact of an intervention or policy. Specific measures of access were lacking in included studies; authors often identified a goal of evaluating the impact of interventions or policies to increase FV access but measured changes in consumption behaviors as a proxy for access.

Measuring FV access seems to be further complicated by a lack of consistent, meaningful, validated instruments; across the studies there was great variability in the ways access was measured. While some studies evaluated the impact of policy change on FV access and consumption, very few looked at population level initiatives or reported on population subgroups to be able to evaluate their impact on children aged five to 18 years. Further, despite the potential benefits of increasing access to FV, only a small number of studies partnered with farms or involved establishing community gardens. We also did not find any studies that evaluated changes in food supply or market inventory, two additional factors that influence access to and consequently consumption of FV.

This review has several methodological and operational limitations. A number of studies that examined knowledge, attitudes, awareness, and general health measures were excluded if they did not also examine either of our primary outcomes of access or consumption. It is possible, that relevant studies that explored our secondary outcomes were missed as a result of these methodological considerations. Operationally, the included studies were limited by the language fluency of the reviewers. Five articles published in Spanish were reviewed and included through titles and abstracts and full text phases, however because the Spanish-speaking reviewers were not available at the data extraction stage we excluded these papers. Data extraction was limited to studies published in English or French. Finally, including participants older than 18 years would have broadened the scope of available literature but the number of studies would not have been manageable for this scoping review. Therefore, the findings of this review are limited to children aged five to 18 years.

## Conclusions

This scoping review sought to identify and map literature that has evaluated the effects of community-based interventions designed to increase FV access and/or consumption among five to 18-year olds. A variety of interventions have been used to support and increase FV consumption. Schools were the most common location for interventions, which were typically multi-faceted, targeted at individuals less than 15 years of age, and delivered by teachers or other school personnel. Additional research on implementing interventions in low- and middle-income countries is warranted based on the limited literature focusing on those populations. Finally, a somewhat narrow field of literature was identified with respect to FV access, suggesting that future research examining interventions to increase FV consumption should include direct outcome measures of FV access. Previously published syntheses revealed a gap in our understanding of the effectiveness of interventions that increase access to FV, since no syntheses that examined access to fruit and vegetables among children were found through our comprehensive literature search. While this scoping review identified several knowledge synthesis products, all were focused on FV consumption. Since consumption is contingent upon access, a focused systematic review that examines these interventions is needed. Such a review should examine and synthesize literature that seeks to increase access through interventions including (but not limited to): influencing FV supply, changing food environments, and enhancing FV sales in cafeterias and grocery stores.

## Competing interests

The authors declare that they have no competing interests.

## Authors’ contributions

RG participated in the conception and methodological design of this review, participated in searching and reviewing studies, and drafted the manuscript. DFL, DC, and LP participated in the conception and methodological design of this review, participated in searching and reviewing, and revised the manuscript. All authors read and approved the final manuscript.

## Pre-publication history

The pre-publication history for this paper can be accessed here:

http://www.biomedcentral.com/1471-2458/12/711/prepub

## Supplementary Material

Additional file 1 Search strategy. Click here for file

Additional file 2 Details of included studies. Click here for file
