# Supplementary material for: Community-based interventions for enhancing access to or consumption of fruit and vegetables among five to 18-year olds: a scoping review
Source: BMC Public Health. 2012 Aug 30;12:711. doi: 10.1186/1471-2458-12-711 (PMC3505745; doi:10.1186/1471-2458-12-711)
Supplement: Additional file 1 — Search strategy. [file 1471-2458-12-711-S1.doc]

**Additional file 1: Search strategy**

**Outcomes**

1. (exp Fruit/ or exp Vegetables/ or exp Vegetable Proteins/ or exp Citrus/ or fruit$.ti,ab. or vegetable$.ti,ab. or citrus.ti,ab or veg.ti,ab.) and (exp Consummatory Behavior/ or exp Feeding Behavior/ or exp Diet/ or exp Food Habits/ or exp Food Preferences/ or exp Health Knowledge, Attitudes, Practice/ or exp Health Behavior/ or Food Supply/)
2. (exp Fruit/ or exp Vegetables/ or exp Vegetable Proteins/ or exp Citrus/) and (consume$1 or consuming or consumption or diet$1 or dietary or eat$1 or ate or eating or intake or exposure or exposed or frequenc$ or frequent).ti,ab.
3. ((fruit$ or vegetable$ or citrus or veg) adj5 (consume$1 or consuming or consumption or diet$1 or dietary or eat$1 or ate or eating or intake$1 or  expos$3 or frequenc$ or frequent)).ti,ab.
4.  (exp Fruit/ or exp Vegetables/ or exp Vegetable Proteins/ or exp Citrus/) and (food environment or food disappearance).ti,ab.
5.  ((fruit$ or vegetable$ or citrus or veg) adj10 (food environment or food disappearance or disappearance method$1)).ti,ab.
6.  (exp Fruit/ or exp Vegetables/ or exp Vegetable Proteins/ or exp Citrus/) and (sales or inventor$).ti,ab.
7.  ((fruit$ or vegetable$ or citrus or veg) adj10 (sales or inventor$)).ti,ab.
8.  ("five a day" or "5 a day").ti,ab.
9.  (exp Fruit/ or exp Vegetables/ or exp Vegetable Proteins/ or exp Citrus/) and (habit$1 or preference$1 or prefer$1 or preferred or choos$ or choice$ or practice$1 or knowledge$ or attitude$ or behavior$1 or behaviour$1).ti,ab.
10. ((fruit$ or vegetable$ or citrus or veg) adj5 (habit$1 or preference$1 or prefer$1 or preferred or choos$ or choice$ or practice$1 or knowledge$ or attitude$ or behavior$1 or behaviour$1)).ti,ab.
11. (exp Fruit/ or exp Vegetables/ or exp Vegetable Proteins/ or exp Citrus/) and (secur$ or insecur$ or sufficien$ or insufficien$ or adequate$ or inadequate$).ti,ab.
12. ((fruit$ or vegetable$ or citrus or veg) adj5 (secur$ or insecur$ or sufficien$ or insufficien$ or adequate$ or inadequate$)).ti,ab.
13. (exp Fruit/ or exp Vegetables/ or exp Vegetable Proteins/ or exp Citrus/) and (access$ or supply$ or supplie$1 or availab$ or inaccess$).ti,ab.
14. ((fruit$ or vegetable$ or citrus or veg) adj5 (access$ or supply$ or supplie$1 or availab$ or inaccess$)).ti,ab.
15. (food secur$ or food insecur$).ti,ab.
16. (food sufficien$ or food insufficien$).ti,ab.
17. or/1-16

**Studies**

18. randomized controlled trial.pt.
19. controlled clinical trial.pt.
20. comparative study.pt.
21. intervention studies/ or evaluation studies/ or program evaluation/
22. random allocation/ or clinical trial/ or single-blind method/ or double-blind method/ or control groups/
23. (randomized or randomised or placebo or randomly or trial or groups).ab.
24. trial.ti.
25. (time adj series).ab,ti.
26. quasi-experiment$.ab,ti.
27. (pre test or pretest or (posttest or post test)).ab,ti.
28. "before and after".ab,ti.
29. controlled before.ab,ti.
30. ((evaluat$ or intervention or interventional) and (control or controlled or study or program$ or comparison or "before and after" or comparative)).ab,ti.
31. (program or programme or secondary analys$).ti,ab.
32. ((intervention or interventional) adj5 evaluat$).ab,ti.
33. or/18-32

**Population/settings terms**

34. (child$3 or boy$1 or girl$ or youth or young person$ or young adult$ or young people or young wom#n or young m#n or youngster* or kid$1 or teen$).ti,ab. or exp adolescent/ or exp child/
35. (family or families or parent$1 or mother$1 or father$1 or mum$1 or dad$1 or grandparent$ or grandmother$ or grandfather$ or sibling$ or brother$ or sister$ or daughter$ or son$1).ti,ab. or family/ or parents/ or fathers/ or mothers/ or single parent/ or siblings/
36. (communit$3 or community-based or local or regional or federal or home$1 or village$1 or town$1 or state wide or province$1 or cities or city or borough$1 or county or counties or rural or urban or neighbourhood$ or neighborhood$ or region$1 or population$1 or suburb$ or slum or slums[I1] ).ti,ab. or "catchment area (health)"/ or population/ or rural population/ or suburban population/ or urban population/ or population groups/ or residence characteristics/ or cities/
37. (school$1 or secondary education or further education or college$1 or student$1 or pupil$1 or schoolchildren).ti,ab. or exp school/
38. (shop$1 or supermarket$ or grocery store$ or grocer$3 or point of purchase$ or market$ or in-store or "in store$" or restaurant$ or cafe$1 or retail$).ti,ab.
39. (workplace$1 or worker$1 or employee$ or work setting$ or worksite$ or work site$ or employer$).ti,ab. or workplace/
40. (job centre$1 or job center$ or public facilit$3 or public service$ or public building$ or post office$).ti,ab. or public facilities/ or swimming pools/
41. (church$ or mosque$ or temple$ or chapel$ or religio3 or centre$ or charit$3 or NGOs or non governmental organi?ation$).ti,ab.
42. (primary care or GP$1 or doctor$1 or general practitioner$1 or family physician$1 or family practice$1).ti,ab. or primary health care/ or family practice/
43. ((leisure or social or recreation$) adj1 (centre$1 or center$1 or facilit$ or club$1)).ti,ab.
44. ((fitness or sport$1 or wellness or health$1) adj1 (centre$1 or center$1 or facilit$ or club$1)).ti,ab.
45. (gym$1 or gymnasia$1 or health spa$1).ti,ab.
46. (cub$1 or scout$1 or scouting or brownies or Girl guides or ranger guide$1 or explorer$1 or rainbow$ or spark$).ti,ab.
47. (youth adj2 (centre$1 or center$1 or facilit$ or club$1)).ti,ab.
48. ethnic groups/ or poverty areas/
49. ((disadvantage$ or marginali#ed or socioeconomic or deprived or deprivation or low income or impoverished or poverty or underserved) adj2 (area$1 or setting$1 or group$1 or population$1)).ti,ab.
50. (ethnic or racial group$1 or ethnicity or minority population$1 or minority group$1).ti,ab.
51. or/34-50
52. 17 and 33 and 51

**Intervention terms**

53. nutrition friendly.ti,ab.
54. (initiative$ or program$ or project$1 or intervention$ or scheme$ or campaign$1).).ti,ab.
55. (access$ or availabl$ or provision or provid$ or offer or supply$ or supplie$1 or distribut$).ti,ab.
56. (promot* or educat*).ti,ab.
57. exp School Health Services/ or exp Community Health Services/ or exp Community Health Nursing/
58. (counsel$ or Advice or advise or advising).ti,ab. or counseling/
58. ((council$1 or coalition$1 or co-op$1 or co-operative$1 or coop$1 or cooperative$1) adj3 (food$1 or fruit$ or vegetable$1 or nutritio$ or grocer$)).ab,ti.
59. (market$ or allotment$ or "grow your own" or homegrown or home grown or kitchen$ or garden$ or social enterprise$ or agriculture or agricultur$ polic$ or land us$3 or land zone$1 or land zoning or urban planning or town planning or edible landscape$1).ti,ab.
60. mass media.ti,ab. or exp Mass Media/
61. social marketing.ti,ab. or exp Social Marketing/
62. information disseminat$.ti,ab. or exp Information Dissemination/
63. media campaign*.ti,ab.
64. trade polic$.ti,ab
65. ("five a day" or "5 a day" or "5-a-day" or "five-a-day").ti,ab.
66. exp Advertising as Topic/ or advert$.ti,ab.
67. exp Public Policy/ or exp Nutrition Policy/
68. ((policy or policies) adj5 (food$1 or fruit$1 or vegetable$1 or nutritio$ or grocer$ or meal$1)).ab,ti.
69. exp Health Education/ or exp Health Promotion/
70. (subsidy or subsidies or subsidi#e$ or economic supplement$ or free or freely or affordab$).ti,ab.
71. (breakfast$ or meal$1 or snack$1 or lunch$).ti,ab.
72. label$.ti,ab. or food labeling/
73. exp Program Evaluation/
74. health planning.ti,ab. or health planning/
75. exp Food Supply/ or food industry/ or food services/
76. Preventive Health Services/
77. exp Dietary Services/
78. or/51-75
79. 17 and 33 and 51 and 78

**Humans filter**

80. animals/ not (humans/ and animals/)
81. 77 not 78
