# Supplementary material for: Community-based interventions for enhancing access to or consumption of fruit and vegetables among five to 18-year olds: a scoping review
Source: BMC Public Health. 2012 Aug 30;12:711. doi: 10.1186/1471-2458-12-711 (PMC3505745; doi:10.1186/1471-2458-12-711)
Supplement: Additional file 2 — Details of included studies. [file 1471-2458-12-711-S2.doc]

Additional file 2: Details of included studies

Author & Title: Multiple articles may be cited that report on results of the same research study. In such cases, the only the primary article is named in the chart.

Study population: Age reported in years

*Categories include: 5-7, 8-10, 11-14, 15-18, parents, teachers, other service providers, general public/ community*

Outcomes:

*Categories include: Access [A]; Consumption [C], Knowledge/Attitudes/Awareness [KA], General Health Measures [GHM], Harms [H]*

| Author(s) & Title | Year | Country | Study population:  Age in years | Outcomes |
| --- | --- | --- | --- | --- |
| **Knowledge syntheses** | | | | |
| Ammerman, A. S., Lindquist, C. H., Lohr, K. N., and Hersey, J. *The efficacy of behavioral interventions to modify dietary fat and fruit and vegetable intake: A review of the evidence* | 2002 | USA | 5-18  Parents,  General public | C |
| Ammerman, A., Lindquist, C., Research Triangle Institute-University of North Carolina Evidence-based Practice Center, United States, and Agency for Healthcare Research and Quality. *The efficacy of interventions to modify dietary behavior related to cancer risk* | 2001 | USA | General public | C |
| Berti, P. R., Krasevec, J., and FitzGerald, S. *A review of the effectiveness of agriculture interventions in improving nutrition outcomes* | 2004 | Canada | General public | A, C |
| Blanchette, L. and Brug, J. *Determinants of fruit and vegetable consumption among 6-12-year-old children and effective interventions to increase consumption* | 2005 | Netherlands | 5-14 | A, C, KA |
| Burchett, H. *Increasing fruit and vegetable consumption among British primary schoolchildren: A review* | 2003 | UK | 5-14 | C |
| Campbell, K., Waters, E., O'Meara, S., and Summerbell, C. *Interventions for preventing obesity in childhood. A systematic review* | 2001 | Australia | 5-14 | C, GHM |
| Ciliska, D., Miles, E., O'Brien, M. A., Turl, C., Tomasik, H. H., Donovan, U., and Beyers, J. *The effectiveness of community interventions to increase fruit and vegetable consumption in people four years of age and older* | 1999 | Canada | 5-18  Parents | C, KA |
| de Sa, J. and Lock, K. *School-based fruit and vegetable schemes: A review of the evidence* | 2007 | UK | 5-18 | C, KA |
| French, S. A. and Stables, G. *Environmental interventions to promote vegetable and fruit consumption among youth in school settings* | 2003 | USA | 5-18 | A, C |
| Hastings, G., Stead, M., McDermott, L., Forsyth, A., MacKintosh, A., and Rayner, M. *Review of research on the effects of food promotion to children* | 2003 | USA | 5-18  Parents | A, C, KA |
| Hingle, M.D., O'Connor, T.M., Dave, J.M., and Baranowski, T. *Parental involvement in interventions to improve child dietary intake: A systematic review* | 2010 | USA | 5-18  Parents | C |
| Howerton, M.W., Bell, B. S., Dodd, K. W., Berrigan, D., Stolzenberg-Solomon, R., and Nebeling, L. *School-based nutrition programs produced a moderate increase in fruit and vegetable consumption: Meta and pooling analyses from 7 studies* | 2007 | USA | 5-14  Other service providers | C, KA |
| Jago, R., Baranowski, T., and Baranowski, J. C. *Fruit and vegetable availability: A micro environmental mediating variable?* | 2007 | USA | 5-14 | A, C |
| Jaime, P. C., and Lock, K. *Do school based food and nutrition policies improve diet and reduce obesity?* | 2009 | Brazil & UK | 5-18 | C |
| Jepson, R., Harris, F., MacGillivray, S., Kearney, N., and Rowa-Dewar, N. *A review of the effectiveness of interventions, approaches and models at individual, community and population levels that are aimed at changing health outcomes through changing knowledge, attitudes and behaviour* | 2006 | UK | 5-18  Parents | C, KA |
| Knai, C., Pomerleau, J., Lock, K., and McKee, M. *Getting children to eat more fruit and vegetables: A systematic review* | 2006 | UK | 5-18 | C, KA |
| Kremers, S. P. J., de Bruijn, G. J., Droomers, M., van Lenthe, F., and Brug, J. (2007). *Moderators of environmental intervention effects on diet and activity in youth* | 2007 | Netherlands | 5-18 | A, C |
| Lissau, I. *Prevention of overweight in the school arena* | 2007 | Denmark | 5-18 | C, KA, GHM |
| McArthur, D. B. *Heart healthy eating behaviors of children following a school-based intervention: A meta-analysis* | 1998 | USA | 8-10 | C, KA, GHM |
| Oldroyd, J., Burns, C., Lucas, P., Haikerwal, A., and Waters, E. *The effectiveness of nutrition interventions on dietary outcomes by relative social disadvantage: A systematic review* | 2008 | Australia | General public | C |
| Pérez-Escamilla, R., Hromi-Fiedler, A., Vega-López, S., Bermúdez-Millán, A., and Segura-Pérez, S. *Impact of peer nutrition education on dietary behaviors and health outcomes among Latinos: A systematic literature review* | 2008 | USA | 5-18  General public | C, KA |
| Pomerleau, J., Lock, K., Knai, C., McKee, M., and European Centre on Health Societies in Transition, London School of Hygiene and Tropical Medicine United Kingdom *Effectiveness of interventions and programmes promoting fruit and vegetable intake in individuals of all ages* | 2005 | UK | 5-18  Parents, General public | C |
| Robinson-O'Brien, R., Story, M., and Heim, S. *Impact of garden-based youth nutrition intervention programs: A review* | 2009 | USA | 5-18 | C, KA |
| Roe, L., Hunt, P., Bradshaw, H., and Rayner, M. *Health promotion interventions to promote healthy eating in the general population: A review* | 1997 | UK | 5-18  Parents | C, KA, GHM |
| Sahay, T. B., Ashbury, F. D., Roberts, M., and Rootman, I. *Effective components for nutrition interventions: A review and application of the literature* | 2006 | Canada | General public | C, KA |
| Shepherd, J., Harden, A., Rees, R., Brunton, G., Garcia, J., Oliver, S., and Oakley, A. *Young people and healthy eating: A systematic review of research on barriers and facilitators* | 2006 | UK | 8-18  Parents | C, KA |
| Summerbell, C. D., Waters, E., Edmunds, L. D., Kelly, S., Brown, T., and Campbell, K. J. *Interventions for preventing obesity in children* | 2005 | UK | 5-14  Parents | A, C, KA, GHM |
| Thomas, H., Ciliska, D., Micucci, S., Wilson-Abra, J., and Dobbins, M. *Effectiveness of physical activity enhancement and obesity prevention programs in children and youth* | 2004 | Canada | 5-18 | C, KA, GHM |
| Van Cauwenberghe, E., Maes, L., Spittaels, H., van Lenthe, F. J., Brug, J., Oppert, J. M., and De Bourdeaudhuij, I. *Effectiveness of school-based interventions in Europe to promote healthy nutrition in children and adolescents: Systematic review of published and 'grey' literature* | 2010 | Netherlands | 5-18 | C, GHM |
| Wall, J., Mhurchu, C. N., Blakely, T., Rodgers, A., and Wilton, J. *Effectiveness of monetary incentives in modifying dietary behavior: A review of randomized, controlled trials* | 2006 | New Zealand | Parents | A, C, KA, GHM |
| **Randomized Controlled Trials** | | | | |
| Baranowski, T., Baranowski, J. C., Cullen, K. W., Thompson, D. I., Nicklas, T., Zakeri, I. F., and Rochon, J. *The Fun, Food, and Fitness Project (FFFP): The Baylor GEMS pilot study* | 2003 | USA | 8-10  Parents | C, GHM |
| Baranowski, T., Baranowski, J., Thompson, D., Buday, R., Jago, R., Griffith, M.J., Islam, N., Nguyen, N., and Watson, K. B. *Video game play, child diet, and physical activity behavior change: A randomized clinical trial* | 2011 | USA | 8-14 | C, GHM |
| Beech, B. M., Klesges, R. C., Kumanyika, S. K., Murray, D. M., Klesges, L., McClanahan, B., Slawson, D., Nunnally, C., Rochon, J., McLain-Allen, B., and Pree-Cary, J. *Child- and parent-targeted interventions: The Memphis GEMS pilot study* | 2003 | USA | 8-10  Parents | C, GHM |
| Chen, J.L., Weiss, S., Heyman, M.B., Cooper, B., and Lustig, R.H. *The efficacy of the web-based childhood obesity prevention program in Chinese American adolescents* | 2011 | USA | 8-18  Parents | C, KA, GHM |
| DeBar, L. L., Ritenbaugh, C., Aickin, M., Orwoll, E., Elliot, D., Dickerson, J., Vuckovic, N., Stevens, V. J., Moe, E., and Irving, L. M. *Youth: A health plan-based lifestyle intervention increases bone mineral density in adolescent girls* | 2006 | USA | 11-18 | C, GHM |
| Epstein, L. H., Gordy, C. C., Raynor, H. A., Beddome, M., Kilanowski, C. K., and Paluch, R. *Increasing fruit and vegetable intake and decreasing fat and sugar intake in families at risk for childhood obesity* | 2001 | USA | 5-14  Parents | C, GHM |
| Faith, M. S., Rose, E., Matz, P. E., Pietrobelli, A., and Epstein, L. H. *Co-twin control designs for testing behavioral economic theories of child nutrition: Methodological note* | 2006 | USA | 5-7 | C |
| Francis, M., Nichols, S. S. D., and Dalrymple, N. *The effects of a school-based intervention programme on dietary intakes and physical activity among primary-school children in Trinidad and Tobago* | 2010 | Trinidad & Tobago | 8-14 | C, KA |
| Fulkerson, J. A., Rydell, S., Kubik, M. Y., Lytle, L., Boutelle, K., Story, M., Neumark-Sztainer, D., Dudovitz, B., and Garwick, A. *Healthy home offerings via the mealtime environment (HOME): Feasibility, acceptability, and outcomes of a pilot study* | 2010 | USA | 8-10  Parents | A, C, KA, GHM |
| Gratton, L., Povey, R., and Clark-Carter, D. *Promoting children's fruit and vegetable consumption: Interventions using the Theory of Planned Behaviour as a framework* | 2007 | UK | 11-18 | C, KA |
| Hendy, H.M., Williams, K.E., and Camise, T.S. *"Kids Choice" school lunch program increases children's fruit and vegetable acceptance* | 2005 | USA | 5-10 | C, KA |
| Jaime, P. C., Machado, F. M., Westphal, M. F., and Monteiro, C. A. *Nutritional education and fruit and vegetable intake: A randomized community trial* | 2007 | Brazil | 5-18  Parents | C |
| Johnston, C. A., Palcic, J. L., Tyler, C., Stansberry, S., Reeves, R. S., and Foreyt, J. P. *Increasing vegetable intake in Mexican-American youth: A randomized controlled trial* | 2011 | USA | 11-14 | C |
| LaPorte, M. R., Gibbons, C. C., and Cross, E. *The effects of a cancer nutrition education program on sixth grade students* | 1989 | USA | 11-14 | C, KA |
| Lubans, D. R., Morgan, P. J., Callister, R., and Collins, C. E. *Effects of integrating pedometers, parental materials, and E-mail support within an extracurricular school sport intervention* | 2009 | Australia | 11-18 | C |
| McKenzie, J., Dixon, L. B., Smiciklas-Wright, H., Mitchell, D., Shannon, B., and Tershakovec, A. *Change in nutrient intakes, number of servings, and contributions of total fat from food groups in 4- to 10-year-old children enrolled in a nutrition education study* | 1996 | USA | 5-10  Parents | C |
| Mihas, C., Mariolis, A., Manios, Y., Naska, A., Arapaki, A., Mariolis-Sapsakos, T., and Tountas, Y. *Evaluation of a nutrition intervention in adolescents of an urban area in Greece: Short- and long-term effects of the VYRONAS study* | 2010 | Greece | 11-14  Parents | C, GHM |
| Patrick, K., Calfas, K. J., Norman, G. J., Zabinski, M. F., Sallis, J. F., Rupp, J., Covin, J., and Cella, J. *Randomized controlled trial of a primary care and home-based intervention for physical activity and nutrition behaviors: PACEplus for adolescents* | 2006 | USA | 11-18  Parents | C, GHM |
| Patrick, K., Sallis, J. F., Prochaska, J. J., Lydston, D. D., Calfas, K. J., Zabinski, M. F., Wilfley, D. E., Saelens, B. E., and Brown, D. R. *A multicomponent program for nutrition and physical activity change in primary care: PACEplus for adolescents* | 2001 | USA | 11-18 | C |
| Pearson, N., Atkin, A. J., Biddle, S. J., and Gorely, T. *A family-based intervention to increase fruit and vegetable consumption in adolescents: A pilot study* | 2010 | UK | 11-14  Parents | C, KA |
| Queral, C. *The impact of a nutrition education program on nutrition knowledge and attitudes, as well as food selection, in a cohort of migrant and seasonal farm worker children* | 2007 | USA | 5-14 | C, KA |
| Rosenberg, D. E., Norman, G. J., Sallis, J. F., Calfas, K. J., and Patrick, K. *Covariation of adolescent physical activity and dietary behaviors over 12 months* | 2007 | USA | 8-18 | C |
| Story, M., Sherwood, N. E., Himes, J. H., Davis, M., Jacobs, D. R., Jr., Cartwright, Y., Smyth, M., and Rochon, J. *An after-school obesity prevention program for African-American girls: The Minnesota GEMS pilot study* | 2003 | USA | 5-7 | A, C, KA, GHM |
| Talvia, S., Rasanen, L., Lagstrom, H., Pahkala, K., Viikari, J., Ronnemaa, T., Arffman, M., and Simell, O. *Longitudinal trends in consumption of vegetables and fruit in Finnish children in an atherosclerosis prevention study (STRIP)* | 2006 | Finland | 5-14  Parents | C |
| Wardle, J., Cooke, L. J., Gibson, E. L., Sapochnik, M., Sheiham, A., and Lawson, M. *Increasing children's acceptance of vegetables: A randomized trial of parent-led exposure* | 2003 | UK | 5-7  Parents | C, KA |
| Warren, J. M., Henry, C. J. K., Lightowler, H. J., Bradshaw, S. M., and Perwaiz, S. *Evaluation of a pilot school programme aimed at the prevention of obesity in children* | 2003 | UK | 5-7 | C, KA, GHM |
| Werch, C., Bian, H., Carlson, J., Moore, M., DiClemente, C., Huang, I., Ames, S., Thombs, D., Weiler, R., and Pokorny, S. *Brief integrative multiple behavior intervention effects and mediators for adolescents* | 2011 | USA | 15-18 | C, KA |
| **Cluster Controlled Studies** | | | | |
| Agozzino, E., Esposito, D., Genovese, S., Manzi, E., and Russo Krauss, P. *Evaluation of the effectiveness of a nutrition education intervention performed by primary school teachers* | 2007 | Italy | 8-10 | C |
| Al Ashfield-Watt, P., Stewart, E. A., and Scheffer, J. A. *A pilot study of the effect of providing daily free fruit to primary-school children in Auckland, New Zealand* | 2009 | New Zealand | 5-14 | C, H |
| Amaro, S., Viggiano, A., Di Costanzo, A., Madeo, I., Baccari, M. E., Marchitelli, E., Raia, M., Viggiano, E., Deepak, S., Monda, M., and De Luca, B. *Kaledo, a new educational board-game, gives nutritional rudiments and encourages healthy eating in children: A pilot cluster randomized trial* | 2002 | Italy | 11-14 | C, KA, GHM |
| Anderson, A., Hetherington, M., Adamson, A., Porteous, L. E. G., Higgins, C., and Foster, E. *The development and evaluation of a novel school based intervention to increase fruit and vegetable intake in children* | 2000 | UK | 5-14 | A, C, KA, GHM |
| Anderson, A. S., Porteous, L. E., Foster, E., Higgins, C., Stead, M., Hetherington, M., Ha, M. A., and Adamson, A. J. *The impact of a school-based nutrition education intervention on dietary intake and cognitive and attitudinal variables relating to fruits and vegetables* | 2005 | UK | 5-14  Teachers | C, KA |
| Angelopoulos, P. D., Milionis, H. J., Grammatikaki, E., Moschonis, G., and Manios, Y. *Changes in BMI and blood pressure after a school based intervention: The CHILDREN study* | 2009 | Greece | 8-14 | C, GHM |
| Ask, A. S., Hernes, S., Aarek, I., Vik, F., Brodahl, C., and Haugen, M. *Serving of free school lunch to secondary-school pupils: A pilot study with health implications* | 2010 | Norway | 11-18 | C, GHM |
| Auld, G. W., Romaniello, C., Heimendinger, J., Hambidge, C., and Hambidge, M. *Outcomes from a school-based nutrition education program alternating special resource teachers and classroom teachers* | 1999 | USA | 5-10 | C, KA |
| Auld, G.W., Romaniello, C., Heimendinger, J., Hambidge, C., and Hambidge, M. *Outcomes from a school-based nutrition education program using resource teachers and cross-disciplinary models* | 1998 | USA | 5-10 | C, KA |
| Baranowski, T., Baranowski, J., Cullen, K. W., deMoor, C., Rittenberry, L., and Hebert, D. *5 a day achievement badge for African-American boy scouts: Pilot outcome results* | 2002 | USA | 8-18 | C, KA |
| Baranowski, T., Baranowski, J., Cullen, K. W., Marsh, T., Islam, N., Zakeri, I., Honess-Morreale, L., and DeMoor, C. *Squire's Quest! Dietary outcome evaluation of a multimedia game* | 2003 | USA | 8-14 | C |
| Baranowski, T., Davis, M., Resnicow, K., Baranowski, J., Doyle, C., Lin, L. S., Smith, M., and Wang, D. T. *Gimme 5 fruit, juice, and vegetables for fun and health: Outcome evaluation* | 2000 | USA | 8-10 | A, C |
| Bates, H. *Promoting healthy eating and active living in schools: A pilot study* | 2010 | Canada | 8-14 | C, GHM |
| Bere, E. *Fruits and vegetables make the mark* | 2004 | Norway | 11-14  Parents | A, C, KA |
| Bere, E., Veierod, M. B., Bjelland, M., and Klepp, K. I. *Outcome and process evaluation of a Norwegian school-randomized fruit and vegetable intervention: Fruits and Vegetables Make the Mark (FVMM)* | 2006 | Norway | 11-14 | A, C, KA |
| Bere, E., Veierod, M. B., and Klepp, K. I. *The Norwegian School Fruit Programme: Evaluating paid vs. no-cost subscriptions* | 2005 | Norway | 11-14 | C |
| Bere, E., Veierod, M. B., Skare, O., and Klepp, K. I. *Free school fruit: Sustained effect three years later* | 2007 | Norway | 11-14 | C |
| Birnbaum, A. S., Lytle, L. A., Story, M., Perry, C. L., and Murray, D. M. *Are differences in exposure to a multicomponent school-based intervention associated with varying dietary outcomes in adolescents?* | 2002 | USA | 11-14 | C, KA |
| Blom-Hoffman, J., Kelleher, C., Power, T. J., and Leff, S. S. *Promoting healthy food consumption among young children: Evaluation of a multi-component nutrition education program* | 2004 | USA | 5-7 | C, KA |
| Blom-Hoffman, J., Wilcox, K.R., Dunn, L., Leff, S. S., and Power, T. J. *Family involvement in school-based health promotion: Bringing nutrition information home* | 2008 | USA | 5-7  Parents | A, C |
| Byrd-Bredbenner, C., O'Connell, L. H., and Shannon, B. *Junior high home economics curriculum: Its effect on students' knowledge, attitude, and behavior* | 1982 | USA | 11-18 | C, KA |
| Coates, T. J., Barofsky, I., and Saylor, K. E. *Modifying the snack food consumption patterns of inner city high school students: The Great Sensations study* | 1985 | USA | 15-18 | C |
| Contento, I. R., Koch, P. A., Lee, H., and Calabrese-Barton, A. *Adolescents demonstrate improvement in obesity risk behaviors after completion of choice, control & change, a curriculum addressing personal agency and autonomous motivation* | 2010 | USA | 11-14 | C, KA |
| Cooke, L.J., Chambers, L.C., Anez, E.V., Croker, H.A., Boniface, D., Yeomans, M.R., and Wardle, J. *Eating for pleasure or profit: The effect of incentives on children's enjoyment of vegetables* | 2011 | UK | 5-7 | C, KA |
| Crockett, S. J., Mullis, R., Perry, C. L., and Luepker, R. V. *Parent education in youth-directed nutrition interventions* | 1989 | USA | 8-10  Parents | A, C, KA |
| Cullen, K.W., Bartholomew, L. K., and Parcel, G. S. *Girl scouting: An effective channel for nutrition education* | 1997 | USA | 8-14 | C, KA |
| Cullen, K., Weber, L., Smalling, A., Thompson, D., Watson, K. B., Reed, D., and Konzelmann, K. *Creating healthful home food environments: Results of a study with participants in the expanded food and nutrition education program* | 2009 | USA | Parents | A |
| Day, M. E., Strange, K. S., McKay, H. A., and Naylor, P. *Action schools! BC -- healthy eating: Effects of a whole-school model to modifying eating behaviours of elementary school children* | 2008 | Canada | 8-10 | C, KA |
| Domel, S. B., Baranowski, T., Davis, H., Thompson, W. O., Leonard, S. B., Riley, P., Baranowski, J., Dudovitz, B., and Smyth, M. *Development and evaluation of a school intervention to increase fruit and vegetable consumption among 4th and 5th grade students* | 1993 | USA | 8-10 | C, KA |
| Dzewaltowski, D. A., Estabrooks, P. A., Welk, G., Hill, J., Milliken, G., Karteroliotis, K., and Johnston, J. A. *Healthy youth places: A randomized controlled trial to determine the effectiveness of facilitating adult and youth leaders to promote physical activity and fruit and vegetable consumption in middle schools* | 2009 | USA | 11-14 | C, KA, GHM |
| Fatohy, I. M., Mounir, G. M., Mahdy, N. H., and El-Deghedi, B. M. *Improving students' knowledge, attitude and practice towards cancer prevention through a health education program. Part II* | 1998 | Egypt | 11-18 | C, KA |
| Foerster, S. B. and Gregson, J. *The California children's 5 a Day Power Play! Campaign: Evaluation study of activities in the school channel* | 1996 | USA | 8-14  Parents | C, KA |
| Fogarty, A. W., Antoniak, M., Venn, A. J., Davies, L., Goodwin, A., Salfield, N., Stocks, J., Britton, J., and Lewis, S. A. *Does participation in a population-based dietary intervention scheme have a lasting impact on fruit intake in young children?* | 2007 | UK | 5-7 | C |
| Foster, G. D., Sherman, S., Borradaile, K. E., Grundy, K. M., Vander Veur, S. S., Nachmani, J., Karpyn, A., Kumanyika, S., and Shults, J. *A policy-based school intervention to prevent overweight and obesity* | 2008 | USA | 8-14  Parents, Teachers | C, GHM, H |
| Fulkerson, J. A., French, S. A., Story, M., Nelson, H., and Hannan, P. J. *Promotions to increase lower-fat food choices among students in secondary schools: Description and outcomes of TACOS (Trying Alternative Cafeteria Options in Schools)* | 2004 | USA | 11-18 | A, KA |
| Gentile, D. A., Welk, G., Eisenmann, J. C., Reimer, R. A., Walsh, D. A., Russell, D. W., Callahan, R., Walsh, M., Strickland, S., and Fritz, K. *Evaluation of a multiple ecological level child obesity prevention program: Switch what you Do, View, and Chew* | 2009 | Ireland | 8-10 | C, GHM |
| German, M. J., Pearce, J., Wyse, B. W., and Hansen, R. G. *A nutrition component for high school health education curriculums* | 1981 | USA | 11-18 | C, KA |
| Gortmaker, S. L., Peterson, K., Wiecha, J., Sobol, A. M., Dixit, S., Fox, M. K., and Laird, N. *Reducing obesity via a school-based interdisciplinary intervention among youth: Planet Health* | 1999 | USA | 8-14 | C, GHM |
| Govula, C. *Culturally appropriate nutrition lessons increased fruit and vegetable consumption in American Indian children* | 2007 | USA | 8-10 | C, KA |
| Green, N. R. and Munrow, S. G. *Evaluating nutrient-based nutrition education by nutrition knowledge and school lunch plate waste* | 1987 | USA | 5-10 | C, KA |
| Haerens, L., Deforche, B., Maes, L., Cardon, G., Stevens, V., and De Bourdeaudhuij, I. *Evaluation of a 2-year physical activity and healthy eating intervention in middle school children* | 2006 | Belgium | 11-14 | C |
| Haire-Joshu, D., Nanney, M. S., Elliott, M., Davey, C., Caito, N., Loman, D., Brownson, R. C., and Kreuter, M. W. *The use of mentoring programs to improve energy balance behaviors in high-risk children* | 2010 | USA | 5-14 | C, KA, GHM |
| Hassapidou, M. N., Fotiadou, E., and Maglara, E. *A nutrition intervention programme for lower secondary schools in Greece* | 1997 | Greece | 11-14 | C, GHM |
| Hazlegrove, S. *Step up MyPyramid - comparing teaching methods for limited resource elementary school children: A pilot study* | 2009 | USA | 8-14 | C, KA |
| He, M., Beynon, C., Sangster Bouck, M., St. Onge, R., Stewart, S., Khoshaba, L., Horbul, B. A., and Chircoski, B. *Impact evaluation of the Northern Fruit and Vegetable Pilot Programme: A cluster-randomised controlled trial* | 2009 | Canada | 5-14 | C, KA |
| Head, M. K. *A nutrition education program at three grade levels* | 1974 | USA | 8-18 | C, KA |
| Hendy, H. M., Williams, K. E., and Camise, T. S. *"Kids Choice" school lunch program increases children's fruit and vegetable acceptance* | 2005 | USA | 5-10 | C, KA |
| Hoffman, J. A., Franko, D. L., Thompson, D. R., Power, T. J., and Stallings, V. A. *Longitudinal behavioral effects of a school-based fruit and vegetable promotion program* | 2010 | USA | 5-7 | C, KA, GHM |
| Hoffman, J. A., Thompson, D. R., Franko, D. L., Power, T. J., Leff, S. S., and Stallings, V. A. *Decaying behavioral effects in a randomized, multi-year fruit and vegetable intake intervention* | 2011 | USA | 5-7 | C, KA, GHM |
| Hopper, C. A., Gruber, M. B., Munoz, K. D., and MacConnie, S. *School-based cardiovascular exercise and nutrition programs with parent participation* | 1996 | USA | 5-14  Parents | C, KA |
| Hoppu, U., Lehtisalo, J., Kujala, J., Keso, T., Garam, S., Tapanainen, H., Uutela, A., Laatikainen, T., Rauramo, U., and Pietinen, P. *The diet of adolescents can be improved by school intervention* | 2010 | Finland | 11-14 | C |
| Horne, P. J., Hardman, C. A., Lowe, C. F., Tapper, K., Le Noury, J., Madden, P., Patel, P., and Doody, M. *Increasing parental provision and children's consumption of lunchbox fruit and vegetables in Ireland: The Food Dudes intervention* | 2009 | UK | 5-14 | C |
| House, J. *Effectiveness of the 5-TODAY program at increasing fruit and vegetable consumption in grade five and six children* | 2005 | Canada | 8-14 | C, KA |
| Jemmott, J. B., Jemmott, L. S., O'Leary, A., Ngwane, Z., Icard, L., Bellamy, S., Jones, S., Landis, J., Heeren, G., Tyler, J. C., and Makiwane, M. B. *Cognitive-behavioural health-promotion intervention increases fruit and vegetable consumption and physical activity among South African adolescents: A cluster-randomised controlled trial* | 2011 | UK | 8-18 | C |
| Kipping, R. R., Jago, R., and Lawlor, D. A. *Diet outcomes of a pilot school-based randomised controlled obesity prevention study with 9-10 year olds in England* | 2010 | USA | 8-10 | C |
| Kirks, B. A., Hendricks, D. G., and Wyse, B. W. *Parent involvement in nutrition education for primary grade students* | 1982 | USA | 5-10 | C, KA |
| Klesges, R. C., Obarzanek, E., Kumanyika, S., Murray, D. M., Klesges, L. M., Relyea, G. E., Stockton, M. B., Lanctot, J. Q., Beech, B. M., McClanahan, B. S., Sherrill-Mittleman, D., and Slawson, D. L. *The Memphis Girls' health Enrichment Multi-site Studies (GEMS): An evaluation of the efficacy of a 2-year obesity prevention program in African American girls* | 2010 | USA | 8-10  Parents | C, GHM |
| Koch, P.A. *A comparison of two nutrition education curricula: Cookshops and food and environment lessons* | 2000 | USA | 5-14 | C, KA |
| Kristal, A. R., Goldenhar, L., Muldoon, J., and Morton, R. F. *Evaluation of a supermarket intervention to increase consumption of fruits and vegetables* | 1997 | USA | General public | A, C |
| Kristjansdottir, A. G., Johannsson, E., and Thorsdottir, I. *Effects of a school-based intervention on adherence of 7-9-year-olds to food-based dietary guidelines and intake of nutrients* | 2010 | Iceland | 5-10 | C |
| Leroy, J. L., Gadsden, P., Rodriguez-Ramirez, S., and De Cossio, T. G. *Cash and in-kind transfers in poor rural communities in Mexico increase household fruit, vegetable, and micronutrient consumption but also lead to excess energy consumption* | 2010 | Mexico | General public | C |
| Lewis, M., Brun, J., Talmage, H., and Rasher, S. *Teenagers and food choices: The impact of nutrition education* [176] | 1988 | USA | 11-18  Teachers | C, KA |
| Lo, E., Coles, R., Humbert, M. L., Polowski, J., Henry, C. J., and Whiting, S. J. *Beverage intake improvement by high school students in Saskatchewan, Canada* | 2008 | Canada | 11-18 | C, KA |
| Lubans, D. R., Morgan, P. J., Callister, R., Collins, C. E., and Plotnikoff, R. C. *Exploring the mechanisms of physical activity and dietary behavior change in the Program X intervention for adolescents* | 2010 | Canada | 11-18 | C, KA |
| Luepker, R. V., Perry, C. L., McKinlay, S. M., Nader, P. R., Parcel, G. S., Stone, E. J., Webber, L. S., Elder, J. P., Feldman, H. A., Johnson, C. C., Kelder, S. H., and Wu, M. for the CATCH Collaborative Group. *Outcomes of a field trial to improve children's dietary patterns and physical activity. The Child and Adolescent Trial for Cardiovascular Health. CATCH collaborative group* | 1996 | USA | 11-14 | C, KA, GHM |
| Lytle, L. A., Murray, D. M., Perry, C. L., Story, M., Birnbaum, A. S., Kubik, M. Y., and Varnell, S. *School-based approaches to affect adolescents' diets: Results from the TEENS study* | 2004 | USA | 11-14  Parents, Other service providers | A, C, KA |
| Mangunkusumo, R. T., Brug, J., de Koning, H. J., van der Lei, J., and Raat, H. *School-based internet-tailored fruit and vegetable education combined with brief counselling increases children's awareness of intake levels* | 2007 | Netherlands | 8-14 | A, C, KA |
| Martens, M. K., van Assema, P., Paulussen, T. G. W. M., Van Breukelen, G., and Brug, J. *Krachtvoer: Effect evaluation of a Dutch healthful diet promotion curriculum for lower vocational schools* | 2008 | Netherlands | 11-14 | C |
| Mauriello, L. M., Ciavatta, M. M., Paiva, A. L., Sherman, K. J., Castle, P. H., Johnson, J. L., and Prochaska, J. M. *Results of a multi-media multiple behavior obesity prevention program for adolescents* | 2010 | USA | 11-18 | C, GHM |
| Moore, L. and Tapper, K. *The impact of school fruit tuck shops and school food policies on children's fruit consumption: A cluster randomised trial of schools in deprived areas* | 2008 | UK | 8-14 | A, C |
| Muth, N. D., Chatterjee, A., Williams, D., Cross, A., and Flower, K. *Making an IMPACT: Effect of a school-based pilot intervention* | 2008 | USA | 8-10 | C, KA, GHM |
| Neumark-Sztainer, D., Story, M., Hannan, P. J., and Rex, J. *New Moves: A school-based obesity prevention program for adolescent girls* | 2003 | USA | 11-18 | C, KA, GHM |
| Nicklas, T. A., Dwyer, J., Mitchell, P., Zive, M., Montgomery, D., Lytle, L., Cutler, J., Evans, M., Cunningham, A., Bachman, K., Nichaman, M., and Snyder, P. *Impact of fat reduction on micronutrient density of children's diets: The CATCH Study* | 1996 | USA | 8-10  Parents | C |
| Nicklas, T. A., Johnson, C. C., Myers, L., Farris, R. P., and Cunningham, A. *Outcomes of a high school program to increase fruit and vegetable consumption: Gimme 5--a fresh nutrition concept for students* | 1998 | USA | 11-18 | C, KA |
| O'Connell, K. M. *Impact of the HEROS (Healthy Eating to Reduce Obesity through Schools) study on healthy food choices and obesity among middle school students in Guilford County (North Carolina) schools* | 2005 | USA | 11-14 | C, KA, GHM |
| Panunzio, M. F., Antoniciello, A., Pisano, A., and Dalton, S. *Nutrition education intervention by teachers may promote fruit and vegetable consumption in Italian students* | 2007 | Italy | 8-14 | C, GHM |
| Parcel, G. S., Simons-Morton, B., O'Hara, N. M., Baranowski, T., and Wilson, B. *School promotion of healthful diet and physical activity: Impact on learning outcomes and self-reported behavior* | 2005 | USA | 5-10 | C, KA |
| Parker, L. and Fox, A. *The Peterborough Schools Nutrition Project: A multiple intervention programme to improve school-based eating in secondary schools* | 2001 | UK | 11-18 | A, C |
| Parmer, S. M., Salisbury-Glennon, J., Shannon, D., and Struempler, B. *School gardens: An experiential learning approach for a nutrition education program to increase fruit and vegetable knowledge, preference, and consumption among second-grade students* | 2009 | USA | 5-10 | C, KA |
| Passmore, S. and Harris, G. *School nutrition action groups and their effect upon secondary school-aged pupils' food choices* | 2005 | UK | 11-18 | A |
| Perry, C. L., Bishop, D. B., Taylor, G. L., Davis, M., Story, M., Gray, C., Bishop, S. C., Mays, R. A. W., Lytle, L. A., and Harnack, L. *A randomized school trial of environmental strategies to encourage fruit and vegetable consumption among children* | 2004 | USA | 5-10 | C |
| Perry, C. L., Bishop, D. B., Taylor, G., Murray, D. M., Mays, R. W., Dudovitz, B. S., Smyth, M., and Story, M. *Changing fruit and vegetable consumption among children: The 5-a-Day Power Plus Program in St. Paul, Minnesota* | 1998 | USA | 8-10 | C, KA |
| Perry, C. L., Luepker, R. V., Murray, D. M., Kurth, C., Mullis, R., Crockett, S., and Jacobs, D. R. Jr. *Parent involvement with children's health promotion: The Minnesota home team* | 1988 | USA | 8-10  Parents | C, KA |
| Perry, C. L., Lytle, L. A., Feldman, H., Nicklas, T., Stone, E., Zive, M., Garceau, A., and Kelder, S. H. *Effects of the Child and Adolescent Trial for Cardiovascular Health (CATCH) on fruit and vegetable intake* | 1998 | USA | 8-10 | C |
| Perry, C. L., Mullis, R., and Maile, M. *Modifying eating behavior of children: A pilot intervention study* | 1985 | USA | 5-10 | C, KA |
| Powers, A. R., Struempler, B. J., Guarino, A., and Parmer, S. M. *Effects of a nutrition education program on the dietary behavior and nutrition knowledge of second-grade and third-grade students* | 2005 | USA | 5-10 | C, KA |
| Prochaska, J. J. and Sallis, J. F. *A randomized controlled trial of single versus multiple health behavior change: Promoting physical activity and nutrition among adolescents* | 2004 | USA | 11-14 | C |
| Quinn, L. J., Horacek, T. M., and Castle, J. *The impact of COOKSHOP on the dietary habits and attitudes of fifth graders* | 2003 | USA | 8-14 | C, KA |
| Radcliffe, B., Ogden, C., Welsh, J., Carroll, S., Coyne, T., and Craig, P. *The Queensland School Breakfast Project: A health promoting schools approach* | 2005 | Australia | 11-14 | C, KA |
| Raju, S., Rajagopal, P., and Gilbride, T. J. *Marketing healthful eating to children: The effectiveness of incentives, pledges, and competitions* | 2010 | USA | 5-14 | C |
| Ransley, J. K., Greenwood, D. C., Cade, J. E., Blenkinsop, S., Schagen, I., Teeman, D., Scott, E., White, G., and Schagen, S. *Does the school fruit and vegetable scheme improve children's diet? A non-randomised controlled trial* | 2007 | UK | 5-7 | C |
| Rees, G., Bakhshi, S., Surujlal-Harry, A., Stasinopoulos, M., and Baker, A. *A computerised tailored intervention for increasing intakes of fruit, vegetables, brown bread and wholegrain cereals in adolescent girls* | 2010 | UK | 11-18 | C, KA |
| Reinaerts, E., De Nooijer, J., Candel, M., and de Vries, N. *Increasing children's fruit and vegetable consumption: Distribution or a multicomponent programme?* | 2007 | Netherlands | 5-14 | C |
| Resnicow, K., Davis, M., Smith, M., Baranowski, T., Lin, L. S., Baranowski, J., Doyle, C., and Wang, D. T. *Results of the TeachWell worksite wellness program* | 1998 | USA | 8-10  Teachers | C, KA, GHM |
| Reynolds, K. D., Bishop, D. B., Chou, C. P., Xie, B., Nebeling, L., and Perry, C. L. *Contrasting mediating variables in two 5-a-day nutrition intervention programs* | 2004 | USA | 8-10 | A, C, KA |
| Reynolds, K. D., Franklin, F. A., Binkley, D., Raczynski, J. M., Harrington, K. F., Kirk, K. A., and Person, S. *Increasing the fruit and vegetable consumption of fourth-graders: Results from the High 5 project* | 2000 | USA | 8-10  Parents | C, KA |
| Ryan, L. *The effect of nutrition education on improving fruit and vegetable consumption of youth* | 1995 | USA | 5-14 | C |
| Sahota, P., Rudolf, M. C., Dixey, R., Hill, A. J., Barth, J. H., and Cade, J. *Randomised controlled trial of primary school based intervention to reduce risk factors for obesity* | 2001 | UK | 5-14  Parents, Teachers, Other service providers | A, C, GHM |
| Shannon, B. and Chen, A. N. *A three-year school-based nutrition education study* | 2010 | USA | 8-10 | C, KA |
| Shemilt, I., Harvey, I., Shepstone, L., Swift, L., Reading, R., Mugford, M., Belderson, P., Norris, N., Thoburn, J., and Robinson, J. *A national evaluation of school breakfast clubs: Evidence from a cluster randomized controlled trial and an observational analysis* | 2004 | UK | 5-14 | C |
| Siega-Riz, A. M., El Ghormli, L., Mobley, C., Gillis, B., Stadler, D., Hartstein, J., Volpe, S. L., Virus, A., and Bridgman, J. *The effects of the HEALTHY study intervention on middle school student dietary intakes* | 2011 | USA | 8-14 | C |
| Smith, H. M. and Justice, C. L. *Effects of nutrition programs on third grade students* | 1979 | USA | 5-10  Parents | C, KA |
| Smolak, L., Levine, M. P., and Schermer, F. *A controlled evaluation of an elementary school primary prevention program for eating problems* | 1998 | USA | 8-14 | C, KA, H |
| Spiegel, S. A. and Foulk, D. *Reducing overweight through a multidisciplinary school-based intervention* | 2006 | USA | 8-10 | C, GHM |
| Taylor, R. W., McAuley, K. A., Barbezat, W., Strong, A., Williams, S. M., and Mann, J. I. *APPLE Project: 2-y findings of a community-based obesity prevention program in primary school age children* | 2007 | New Zealand | 5-10 | C, GHM |
| te Velde, S. J., Brug, J., Wind, M., Hildonen, C., Bjelland, M., Perez-Rodrigo, C., and Klepp, K. I. *Effects of a comprehensive fruit- and vegetable-promoting school-based intervention in three European countries: The Pro Children Study* | 2008 | Norway | 8-14 | C |
| Thompson, D., Baranowski, T., Baranowski, J., Cullen, K., Jago, R., Watson, K., and Liu, Y. *Boy scout 5-a-day badge: Outcome results of a troop and internet intervention* | 2009 | USA | 8-18 | A, C, KA, GHM |
| Thompson, V., Cullen, K. W., Watson, K. B., and Zakeri, I. *The increased availability and marketing of fruit, juice, and vegetables to middle school students increases consumption* | 2007 | USA | 11-14 | C |
| Weaver, M., Poehlitz, M., and Hutchison, S. *5 a day for low-income families: Evaluation of an advertising campaign and cooking events* | 1999 | USA | 5-7  Parents, General public | C |
| White, A. A. and Skinner, J. D. *Can goal setting as a component of nutrition education affect behavior change among adolescents?* | 1988 | USA | 11-18 | C, KA |
| Wilson, D. K., Friend, R., Teasley, N., Green, S., Reaves, I. L., and Sica, D. A. *Motivational versus social cognitive interventions for promoting fruit and vegetable intake and physical activity in African American adolescents* | 2002 | USA | 11-18 | C, KA |
| Wind, M., Bjelland, M., Pérez-Rodrigo, C., te Velde, S. J., Hildonen, C., Bere, E., Klepp, K. I., and Brug, J. *Appreciation and implementation of a school-based intervention are associated with changes in fruit and vegetable intake in 10- to 13-year old schoolchildren: The Pro Children study* | 2006 | Norway, Spain & Netherlands | 8-14 | C |
| Winett, R. A., Roodman, A. A., Winett, S. G., Bajzek, W., Rovniak, L. S., and Whiteley, J. A. *The effects of the Eat4Life internet-based health behavior program on the nutrition and activity practices of high school girls* | 1999 | USA | 11-18 | C |
| **Quasi-Experimental** | | | | |
| Auld, G. W., Romaniello, C., Heimendinger, J., Hambidge, C., and Hambidge, M. (1998). *Outcomes from a school-based nutrition education program using resource teachers and cross-disciplinary models* | 1998 | USA | 5-14  Parents, Teachers | C, KA |
| Bates, N. J. *An evaluation of a stage of change nutrition intervention in Latino families with young children* | 2001 | USA | 5-7  Parents | C |
| Bell, C. G. and Lamb, M. W. *Nutrition education and dietary behavior of fifth graders* | 1973 | USA | 8-14 | C, KA |
| Boaz, A., Ziebland, S., Wyke, S., and Walker, J. *A 'five-a-day' fruit and vegetable pack for primary school children. Part II: Controlled evaluation in two Scottish schools* | 1998 | UK | 5-10 | C |
| Cade, J. and Lambert, H. *Evaluation of the effect of the removal of the family income supplement (FIS) free school meal on the food intake of secondary schoolchildren* | 1991 | UK | 11-18 | C |
| Casazza, K. *A computer based approach to improve the dietary and physical activity patterns of a diverse group of adolescents* | 2006 | USA | 11-18 | C, KA, GHM |
| Craven, K., Moore, J., Swart, A., Keene, A., and Kolasa, K. *School-based nutrition education intervention: Effect on achieving a healthy weight among overweight ninth-grade students* | 2011 | USA | 11-18 | C, GHM |
| Covelli, M. M. *Efficacy of a school-based intervention on blood pressure and cortisol levels of African American adolescents* | 2000 | USA | 15-18 | C, GHM |
| Cullen, K. W., Watson, K., Baranowski, T., Baranowski, J. H., and Zakeri, I. *Squire's Quest: Intervention changes occurred at lunch and snack meals* | 2005 | USA | 8-10 | C |
| Cullen, K. W. and Zakeri, I. *Fruits, vegetables, milk, and sweetened beverages consumption and access to a la carte/snack bar meals at school* | 2004 | USA | 8-14 | C |
| Cummins, S., Petticrew, M., Higgins, C., Findlay, A., and Sparks, L. *Large scale food retailing as an intervention for diet and health: Quasi-experimental evaluation of a natural experiment* | 2005 | UK | General public | C, GHM |
| Day, L. L. and Rodriguez, E. C. *Impact of a field trip to a health museum on children's health-related behaviors and perceived control over illness* | 2002 | USA | 8-14 | C |
| Di Noia, J., Contento, I. R., and Prochaska, J. O. *Computer-mediated intervention tailored on transtheoretical model stages and processes of change increases fruit and vegetable consumption among urban African-American adolescents* | 2008 | USA | 11-14 | C, KA |
| Edwards, C. S. and Hermann, J. R. *Piloting a cooperative extension service nutrition education program on first-grade children's willingness to try foods containing legumes* | 2011 | USA | 5-7 | C |
| Eriksen, K., Haraldsdóttir, J., Pederson, R., and Flyger, H. V. *Effect of a fruit and vegetable subscription in Danish schools* | 2003 | Denmark | 5-10 | C |
| Fahlman, M. M., Dake, J. A., McCaughtry, N., and Martin, J. *A pilot study to examine the effects of a nutrition intervention on nutrition knowledge, behaviors, and efficacy expectations in middle school children* | 2008 | USA | 11-14 | C, KA |
| Foerster, S. B., Gregson, J., Beall, D. L., Hudes, M., Magnuson, H., Livingston, S., Davis, M. A., Joy, A. B. J., and Garbolino, T. *The California children's 5 a Day Power Play! Campaign: Evaluation of a large-scale social marketing initiative* | 1998 | USA | 8-10 | C, KA |
| Friel, S., Kelleher, C., Campbell, P., and Nolan, G. (1999). *Evaluation of the Nutrition Education at Primary School (NEAPS) programme* | 1999 | UK | 8-10  Parents | C, KA |
| Georgiou, C. *The effect of nutrition education on third graders' school lunch consumption in a school offering food pyramid choice menus* | 1998 | USA | 8-10 | C |
| Gortmaker, S. L., Cheung, L. W. Y., Peterson, K. E., Chomitz, G., Cradle, J. H., Dart, H., Fox, M. K., Bullock, R. B., Sobol, A. M., Colditz, G., Field, A. E., and Laird, N. *Impact of a school-based interdisciplinary intervention on diet and physical activity among urban primary school children: Eat well and keep moving* | 1999 | USA | 8-10 | C, KA, GHM |
| Gosliner, W. A., James, P., Yancey, A. K., Ritchie, L., Studer, N., and Crawford, P. B. *Impact of a worksite wellness program on the nutrition and physical activity environment of child care centers* | 2010 | USA | 5-18  Other service providers | A |
| Gribble, L. S., Falciglia, G., Davis, A. M., and Couch, S. C. *A curriculum based on social learning theory emphasizing fruit exposure and positive parent child-feeding strategies: A pilot study* | 2003 | USA | 8-14 | C, KA |
| Hendy, H. M., Williams, K. E., and Camise, T. S. *Kid's Choice Program improves weight management behaviors and weight status in school children* | 2011 | USA | 5-10 | A, C, KA, GHM |
| Hoddinott, J. and Wiesmann, D. *The impact of conditional cash transfer programs on food consumption in Honduras, Mexico, and Nicaragua* | 2010 | Honduras, Mexico, Nicaragua | General public | A |
| Hollar, D., Messiah, S. E., Lopez-Mitnik, G., Hollar, T. L., and Agatston, A. S. *Effect of a school-based obesity intervention on weight and blood pressure in 6-13 year olds* | 2010 | USA | 5-14  Parents, Teachers | A, GHM |
| Horne, P. J., Tapper, K., Lowe, C. F., Hardman, C. A., Jackson, M. C., and Woolner, J. *Increasing children's fruit and vegetable consumption: A peer-modeling and rewards-based intervention* | 2004 | UK | 5-14 | C |
| Jacob, T. *Evaluation of food and fun for everyone: A nutrition education program for third and fourth grade students* | 2009 | USA | 8-10 | C |
| Jamelske, E., Bica, L. A., McCarty, D. J., and Meinen, A. *Preliminary findings from an evaluation of the USDA fresh fruit and vegetable program in Wisconsin schools* | 2008 | USA | 8-14 | C, KA |
| Jimenez, M. M., Receveur, O., Trifonopoulos, M., Kuhnlein, H., Paradis, G., and Macaulay, A. C. *Comparison of the dietary intakes of two different groups of children (grades 4 to 6) before and after the Kahnawake Schools Diabetes Prevention Project* | 2003 | Canada | 5-14 | C, GHM |
| Kelder, S., Hoelscher, D. M., Barroso, C. S., Walker, J. L., Cribb, P., and Hu, S. *The CATCH Kids Club: A pilot after-school study for improving elementary students' nutrition and physical activity* | 2005 | USA | 5-14 | C, KA |
| Liquori, T., Koch, P. D., Contento, I. R., and Castle, J. *The cookshop program: Outcome evaluation of a nutrition education program linking lunchroom food experiences with classroom cooking experiences* | 1998 | USA | 5-14 | C, KA |
| Long, J. D. *The effects of a school-based nutrition education intervention on self-efficacy for healthy eating, usual food choices, dietary knowledge, and fruit, vegetable, and fat consumption in adolescents* | 2001 | USA | 11-18 | C, KA |
| Long, J. D. and Stevens, K. R. *Using technology to promote self-efficacy for healthy eating in adolescents* | 2004 | USA | 11-18 | C, KA |
| Manios, Y., Moschandreas, J., Hatzis, C., and Kafatos, A. *Health and nutrition education in primary schools of Crete: Changes in chronic disease risk factors following a 6-year intervention programme* | 2002 | Greece | 5-14 | C, KA, GHM |
| Matvienko, O. *Impact of a nutrition education curriculum on snack choices of children ages six and seven years* | 2007 | USA | 5-7 | C |
| McAleese, J. D. and Rankin, L. L. *Garden-based nutrition education affects fruit and vegetable consumption in sixth-grade adolescents* | 2007 | USA | 8-14 | C |
| Morgan, P. J., Warren, J. M., Lubans, D. R., Saunders, K. L., Quick, G. I., and Collins, C. E. *The impact of nutrition education with and without a school garden on knowledge, vegetable intake and preferences and quality of school life among primary-school students* | 2010 | Australia | 11-14 | C, KA |
| Olvera, N., Bush, J. A., Sharma, S. V., Knox, B. B., Scherer, R. L., and Butte, N. F. *BOUNCE: A community-based mother-daughter healthy lifestyle intervention for low-income Latino families* | 2010 | USA | 5-14  Parents | C |
| Perry, C. L., Zauner, M., Oakes, J. M., Taylor, G., and Bishop, D. B. *Evaluation of a theater production about eating behavior of children* | 2002 | USA | 5-14 | C, KA |
| Raghunatha Rao, D., Vijayapushpam, T., Subba Rao, G. M., Antony, G. M., and Sarma, K. V. R. *Dietary habits and effect of two different educational tools on nutrition knowledge of school going adolescent girls in Hyderabad, India* | 2007 | India | 11-14 | C, KA |
| Ratcliffe, M. M., Merrigan, K. A., Rogers, B. L., and Goldberg, J. P. *The effects of school garden experiences on middle school-aged students' knowledge, attitudes, and behaviors associated with vegetable consumption* | 2011 | USA | 11-14 | C, KA |
| Russ, C. R., Tate, D. F., Whiteley, J. A., Winett, R. A., Winett, S. G., and Pfleger, J. *The effects of an innovative WWW-based health behavior program on the nutritional practices of tenth grade girls: Preliminary report on the Eat4Life Program* | 1998 | USA | 15-18 | C |
| Schagen, S., Blenkinsop, S., Schagen, I., Scott, E., Teeman, D., White, G., Ransley, J., Cade, J., and Greenwood, D. *Evaluation of the school fruit and vegetable pilot scheme: Final report* | 2005 | UK | 5-10 | C, KA |
| Schwartz, M. B. (2007). *The influence of a verbal prompt on school lunch fruit consumption: A pilot study* | 2007 | USA | 5-10 | C |
| Schwartz, R. P., Hamre, R., Dietz, W. H., Wasserman, R. C., Slora, E. J., Myers, E. F., Sullivan, S., Rockett, H., Thoma, K. A., Dumitru, G., and Resnicow, K. A. *Office-based motivational interviewing to prevent childhood obesity: A feasibility study* | 2007 | USA | 5-7 | C, GHM |
| Shannon, B., Graves, K., and Hart, M. *Food behavior of elementary school students after receiving nutrition education* | 1982 | USA |  | A, C |
| Simons-Morton, B. G., Parcel, G. S., Baranowski, T., Forthofer, R., and O'Hara, N. M. *Promoting physical activity and a healthful diet among children: Results of a school-based intervention study* | 1991 | USA | 5-10 | C |
| Singhal, N., Misra, A., Shah, P., and Gulati, S. *Effects of controlled school-based multi-component model of nutrition and lifestyle interventions on behavior modification, anthropometry and metabolic risk profile of urban Asian Indian adolescents in North India* | 2010 | India | Parents, Teachers | C, KA, GHM |
| Tanner, A., Duhe, S., Evans, A., and Condrasky, M. *Using student-produced media to promote healthy eating: A pilot study on the effects of a media and nutrition intervention* | 2008 | United States (USA) | 8-10 | A, C, KA |
| Tak, N. I., te Velde, S. J., and Brug, J. *Long-term effects of the Dutch Schoolgruiten Project--promoting fruit and vegetable consumption among primary-school children* | 2009 | Netherlands | 8-14 | A, C, KA |
| Vargas, I. C. S., Sichieri, R., Sandre-Pereira, G., and da Veiga, G. V. *Evaluation of an obesity prevention program in adolescents of public schools* | 2011 | Brazil | 11-18 | C, GHM |
| Wagner, J. L. *The relationship of parent and child food choices: Influences of a supermarket intervention* | 1991 | Canada | 8-18  Parents | A, C |
| Walsh, C. M., Dannhauser, A., and Joubert, G. *Impact of a nutrition education programme on nutrition knowledge and dietary practices of lower socioeconomic communities in the Free State and Northern Cape* | 2003 | South Africa | General public | A, C, KA |
| White, G. *Evaluation of the school fruit and vegetable pilot scheme* | 2006 | UK | 5-7 | C, KA |
| Williams, J. E. *Social support and adolescent nutrition behaviors in African-American families* | 2004 | USA | 11-18 | C, KA |
| **Controlled Time Series** | | | | |
| Forneris, T., Fries, E., Meyer, A., Buzzard, M., Uguy, S., Ramakrishnan, R., Lewis, C., and Danish, S. *Results of a rural school-based peer-led intervention for youth: Goals for health* | 2010 | USA | 8-14 | C, KA |
| Gorely, T., Nevill, M. E., Morris, J. G., Stensel, D. J., and Nevill, A. *Effect of a school-based intervention to promote healthy lifestyles in 7-11 year old children* | 2009 | United Kingdom (UK) | 5-14 | C, KA, GHM |
| Somerset, S. and Markwell, K. *Impact of a school-based food garden on attitudes and identification skills regarding vegetables and fruit: A 12-month intervention trial* | 2009 | Australia | 8-14 | C, KA |
| **Before-After Studies (no control)** | | | | |
| Belansky, E. S., Cutforth, N., Delong, E., Litt, J., Gilbert, L., Scarbro, S., Beatty, B., Romaniello, C., Brink, L., and Marshall, J. A. *Early effects of the federally mandated local wellness policy on school nutrition environments appear modest in Colorado's rural, low-income elementary schools* | 2010 | USA | 5-18  Other service providers | A |
| Bere, E. and Klepp, K. I. *Free vs. paid school fruit: Big difference with respect to social inequality* | 2006 | Norway | 11-18 | C, GHM |
| Birmingham, B., Shultz, J. A., and Edlefsen, M. *Evaluation of a five-a-day recipe booklet for enhancing the use of fruits and vegetables in low-income households* | 2004 | USA | Parents | C |
| Brown, B. J. and Hermann, J. R. *Gem no. 395. Cooking classes increase fruit and vegetable intake and food safety behaviors in youth and adults* | 2005 | USA | 11-14 | C |
| Caldwell, E. M., Miller Kobayashi, M., DuBow, W. M., and Wytinck, S. M. *Perceived access to fruits and vegetables associated with increased consumption* | 2009 | USA | General public | A, C |
| Canavera, M., Sharma, M., and Murnan, J. *Development and pilot testing a social cognitive theory-based intervention to prevent childhood obesity among elementary students in rural Kentucky* | 2008 | USA | 5-14 | C, KA |
| Cassady, D., Vogt, R., Oto-Kent, D., Mosley, R., and Lincoln, R. *The power of policy: A case study of healthy eating among children* | 2006 | USA | 5-14 | A, C |
| Centers for Disease Control and Prevention. *Evaluation of a fruit and vegetable distribution program--Mississippi, 2004-05 school year* | 2006 | USA | 8-18 | C, KA |
| Clawson, B., Sumner, S. K., and McPherson, E. P. *Effectiveness of a school-based community approach to nutrition education for adolescents* | 1984 | USA | 11-18  Teachers, General public | C, KA |
| Colby, S. E. *The development of peer-led youth theater as a nutrition education tool to promote the healthy traditional Latino diet* | 2005 | USA | 8-14 | C, KA, GHM |
| Contento, I. R., Koch, P. A., Lee, H., Sauberli, W., and Calabrese-Barton, A. *Enhancing personal agency and competence in eating and moving: Formative evaluation of a middle school curriculum--Choice, Control, and Change* | 2007 | USA | 11-14 | C, KA |
| Coyle, K. K., Potter, S., Schneider, D., May, G., Robin, L. E., Seymour, J., and Debrot, K. *Distributing free fresh fruit and vegetables at school: Results of a pilot outcome evaluation* | 2009 | USA | 8-18 | C, KA |
| Cullen, K.W., Hartstein, J., Reynolds, K. D., Vu, M., Resnicow, K., Greene, N., White, M. A., and For the Studies to Treat or Prevent Pediatric Type 2 Diabetes Prevention Study Group. *Improving the school food environment: Results from a pilot study in middle schools* | 2007 | USA | 11-14 | A |
| Cullen, K. W. and Thompson, D. *Feasibility of an 8-week African American web-based pilot program promoting healthy eating behaviors: Family Eats* | 2008 | USA | 8-14  Parents | A |
| Cullen, K. W. and Watson, K. B. *The impact of the Texas public school nutrition policy on student food selection and sales in Texas* | 2009 | USA | 5-18 | A, C |
| Cullen, K. W., Watson, K., and Zakeri, I. *Improvements in middle school student dietary intake after implementation of the Texas Public School Nutrition Policy* | 2008 | USA | 11-14 | C |
| Cullen, K. W., Watson, K. B., Zakeri, I., Baranowski, T., and Baranowski, J. H. *Achieving fruit, juice, and vegetable recipe preparation goals influences consumption by 4th grade students* | 2007 | USA | 8-10 | C, KA |
| Cullen, K. W., Watson, K., Zakeri, I., and Ralston, K. *Exploring changes in middle school student lunch consumption after local school food service policy modifications* | 2006 | USA | 8-14 | A, C |
| Cullen, K. W., Zakeri, I., Pryor, E. W., Baranowski, T., Baranowski, J., and Watson, K. *Goal setting is differentially related to change in fruit, juice, and vegetable consumption among fourth-grade children* | 2004 | USA | 8-10 | C, KA |
| DelCampo, D., Baca, J. S., Jimenez, D., Sanchez, P. R., and DelCampo, R. *Just Be It! Healthy and fit increases fifth graders' fruit and vegetable intake, physical activity, and nutrition knowledge* | 2011 | USA | 8-14  Parents, Teachers | C, KA |
| Engels, H. J., Gretebeck, R. J., Gretebeck, K. A., and Jimenez, L. *Promoting healthful diets and exercise: Efficacy of a 12-week after-school program in urban African Americans* | 2005 | USA | 8-18 | C, GHM |
| Freedman, M. R. and Nickell, A. *Impact of after-school nutrition workshops in a public library setting* | 2010 | USA | 8-14 | C |
| French, S. A., Story, M., Jeffery, R. W., Snyder, P., Eisenberg, M., Sidebottom, A., and Murray, D. *Pricing strategy to promote fruit and vegetable purchase in high school cafeterias* | 1997 | USA | 11-18 | A |
| Greenwood, B., Ralston, P. A., Young-Clark, I., Cornille, T., Brown, L. L., Davis, K. E., Salley, T. J., Goehrig, M. H., Mullins, A. P., and Gaskins, D. J. *Nutrition education initiative: A school-based program to promote healthy eating practices of preadolescents* | 2009 | USA | 11-14 | C |
| Goldberg, J. P., Collins, J. J., Folta, S. C., McLarney, M. J., Kozower, C., Kuder, J., Clark, V., and Economos, C. D. *Retooling food service for early elementary school students in Somerville, Massachusetts: The Shape Up Somerville experience* | 2009 | USA | 5-10 | A, KA |
| Grainger, C., Senauer, B., and Runge, C. F. *Nutritional improvements and student food choices in a school lunch program* | 2007 | USA | 11-18 | C |
| Haroun, D., Harper, C., Wood, L., and Nelson, M. *The impact of the food-based and nutrient-based standards on lunchtime food and drink provision and consumption in primary schools in England* | 2011 | UK | 5-14 | A, C, GHM |
| Heim, S., Bauer, K. W., Stang, J., and Ireland, M. *Can a community-based intervention improve the home food environment? Parental perspectives of the influence of the delicious and nutritious Garden* | 2011 | USA | 8-14 | A, KA |
| Heim, S., Stang, J., and Ireland, M. *A garden pilot project enhances fruit and vegetable consumption among children* | 2009 | USA | 8-14 | A, C, KA |
| Hermann, J. R., Parker, S. P., Brown, B. J., Siewe, Y. J., Denney, B. A., and Walker, S. J. *After-school gardening improves children's reported vegetable intake and physical activity* | 2006 | USA | 5-14 | C |
| Horne, P. J., Lowe, C. F., Fleming, P. F., and Dowey, A. J. *An effective procedure for changing food preferences in 5-7-year-old children* | 1995 | UK | 5-7 | C |
| Howison, D., Neidermyer, F., and Shortridge, R. *Field testing a fifth-grade nutrition education program designed to change food-selection behavior* | 1988 | USA | 8-14 | C, KA |
| Jones, J. H., Jr. *Evaluation of the Louisiana nutrition education program* | 1970 | USA | 5-14 | C, KA |
| Koch, S., Waliczek, T. M., and Zajicek, J. M. *The effect of a summer garden program on the nutritional knowledge, attitudes, and behaviors of children* | 2006 | USA | 5-14 | C, KA |
| Kuczmarski, M. F. and Aljadir, L. *Gem no. 364. Using food calendars to self-monitor: Got 5? Nutrition for Kids program* | 2003 | USA | 5-10 | C |
| Latimer, M. *The role of flavor-flavor conditioning and sensory-based, vegetable-themed education in increasing vegetable consumption in elementary school-aged children* | 2009 | USA | 5-14 | C, KA |
| Laurence, S., Peterken, R., and Burns, C. *Fresh Kids: The efficacy of a health promoting schools approach to increasing consumption of fruit and water in Australia* | 2007 | Australia | 5-14 | C |
| Lautenschlager, L. and Smith, C. *Understanding gardening and dietary habits among youth garden program participants using the theory of planned behavior* | 2007 | USA | 8-18 | C, KA |
| Lee, A., Ho, M., and Keung, V. *Healthy school as an ecological model for prevention of childhood obesity* | 2010 | China | 8-14 | A, C, KA |
| Lindholm, B. W., Touliatos, J., and Wenberg, M. F. *Predicting changes in nutrition knowledge and dietary quality in ten to thirteen-year-olds following a nutrition education program* | 1984 | USA | 8-14 | C, KA |
| Lineberger, S. E. and Zajicek, J. M. *School gardens: Can a hands-on teaching tool affect students' attitudes and behaviors regarding fruit and vegetables?* | 2000 | USA | 5-14 | C, KA |
| Long, J. D., Armstrong, M. L., Amos, E., Shriver, B., Roman-Shriver, C., Feng, D., Harrison, L., Luker, S., Nash, A., and Blevins, M. W. *Pilot using World Wide Web to prevent diabetes in adolescents* | 2006 | USA | 11-18 | C, GHM |
| Lowe, C. F., Horne, P. J., Tapper, K., Bowdery, M., and Egerton, C. *Effects of a peer modelling and rewards-based intervention to increase fruit and vegetable consumption in children* | 2004 | UK | 5-14 | C, KA |
| Lowe, F. and Horne, P. *Food Dudes. Increasing children's consumption of fruit and vegetables. Changing the nation's diet: A programme to increase children's consumption of fruit and vegetables* | 2007 | UK | 5-7 | C |
| Maddock, J., Takeuchi, L., Netta, B., Tanaka, C., Irvin, L., Matsuoka, C., and Wood, B. *Evaluation of a statewide program to reduce chronic disease: The Healthy Hawaii Initiative, 2000-2004* | 2006 | USA | General public | A, C, KA |
| Martens, M., van Assema, P., Knibbe, R., Engels, R. C., and Brug, J. *Family environmental factors do not explain differences in the behavioral effect of a healthy diet promotion program in lower vocational schools among 12- to 14-year-old adolescents* | 2010 | Netherlands | 11-18 | C |
| Matusky, J., Gilboy, M., and Anderson, K. *A collaborative model for nutrition education in elementary schools changes the fruit and vegetable consumption of second grade students* | 2009 | USA | 5-10 | C |
| McCormick, A., Kattelmann, K., Ren, C., Richards, A., and Wells, K. *"Fun fruit and veggie event" enhances acceptance of fruits and vegetables in school-aged children* | 2009 | USA | 5-18 | C, KA |
| Medina, J. *A dose-response analysis of a school-based nutrition intervention in middle school children* | 2009 | USA | 11-14 | C, KA |
| Mozaffarian, R. S., Wiecha, J. L., Roth, B. A., Nelson, T. F., Lee, R. M., and Gortmaker, S. L. *Impact of an organizational intervention designed to improve snack and beverage quality in YMCA after-school programs* | 2010 | USA | 5-14 | A |
| Mullally, M. L., Taylor, J. P., Kuhle, S., Bryanton, J., Hernandez, K. J., MacLellan, D. L., McKenna, M. L., Gray, R. J., and Veugelers, P. J. *A province-wide school nutrition policy and food consumption in elementary school children in Prince Edward Island* | 2010 | Canada | 8-14 | C |
| Nelson, M., Lowes, K., Hwang, V., and members of the UK Nutrition Group, School Meals Panel, Department for Education and Skills. *The contribution of school meals to food consumption and nutrient intakes of young people aged 4-18 years in England* | 2007 | UK | 5-18 | C |
| Panunzio, M. F., Antoniciello, A., Cela, E. P., Ferguson, L. R., Bucci, E., Petracca, L., Bisceglia, R., D'Ambrosio, P., Buccinotti, M. C., Romagnolo, G., D'Aprile, A. P., Carella, F., and Ugolini, G. *15-week long school-based nutritional education program to promote Italian primary schoolchildren's fruit and vegetable intake* | 2010 | Italy | 5-14 | C |
| Perez-Rodrigo, C. and Aranceta, J. *Nutrition education for schoolchildren living in a low-income urban area in Spain* | 1997 | Spain | 8-14 | A, C, KA |
| Perrin, E. M., Jacobson Vann, J. C., Benjamin, J. T., Skinner, A. C., Wegner, S., and Ammerman, A. S. *Use of a pediatrician toolkit to address parental perception of children's weight status, nutrition, and activity behaviors* | 2010 | USA | 5-14  Parents | C, GHM |
| Resnicow, K., Yaroch, A. L., Davis, A., Wang, D. T., Carter, S., Slaughter, L., Coleman, D., and Baranowski, T. *GO GIRLS!: Results from a nutrition and physical activity program for low-income, overweight African American adolescent females* | 2000 | USA | 11-18 | C, KA, GHM |
| Rinderknecht, K. and Smith, C. *Social cognitive theory in an after-school nutrition intervention for urban Native American youth* | 2004 | USA | 8-14  Parents | C, KA |
| Scampini, R., Bandini, L., Curtin, C., Gleason, J., Must, A., Maslin, M., and Fleming, R. *Changes in fruit, vegetable and sweetened beverage intake in adolescents and young adults with down syndrome participating in a parent supported weight reduction program* | 2008 | USA | 11-18  Parents | C |
| Seo, D. *Comparison of school food policies and food preparation practices before and after the local wellness policy among Indiana high schools* | 2009 | USA | 11-18 | A |
| Slusser, W. M., Cumberland, W. G., Browdy, B. L., Lange, L., and Neumann, C. *A school salad bar increases frequency of fruit and vegetable consumption among children living in low-income households* | 2007 | USA | 5-14 | C |
| Spoon, M. D., Benedict, J., Leontos, C., and Krelle-Zepponi, N. *Increasing fruit and vegetable consumption among middle school students: Implementing the 5-a-Day program* | 1998 | USA. | 8-18  Parents | C, KA |
| Stawinski, M., Jaworowicz-Szczepaniak, M., Adamek, R., and Maksymiuk, T. *Results of introducing a health education programme in a Poznan kindergarten: A preliminary study* | 2008 | Poland | 5-7 | C |
| Teeman, D., Blenkinsop, S., Ransley, J., Schagen, I., Schagen, S., Scott, E., and White, G. *Evaluation of the Big Lottery Fund's National School Fruit and Vegetable Scheme: Second interim report* | 2004 | UK | 5-7 | C, KA |
| Trevino, R. P., Pugh, J. A., Hernandez, A. E., Menchaca, V. D., Ramirez, R. R., and Mendoza, M. *Bienestar: A diabetes risk-factor prevention program* | 1998 | USA | 8-10  Parents, Teachers | C, KA, GHM |
| Tse, M. M. Y. and Yuen, D. T. W. *Effects of providing a nutrition education program for teenagers: Dietary and physical activity patterns* | 2009 | China | 11-14 | C |
| Walker, S. J. *Effect of an after school education and gardening program on nutrition and physical activity behaviors in school age youth, grades 3-8* | 2006 | USA | 5-14 | C |
| Wang, M. C., Rauzon, S., Studer, N., Martin, A. C., Craig, L., Merlo, C., Fung, K., Kursunoglu, D., Shannguan, M., and Crawford, P. *Exposure to a comprehensive school intervention increases vegetable consumption* | 2010 | USA | 8-14 | C, KA |
| Wright, W. and Rowell L. *Examining the effect of gardening on vegetable consumption among youth in kindergarten through fifth grade* | 2010 | USA | 5-14 | A, C |
| Wrigley, N. *Assessing the impact of improved retail access on diet in a 'food desert': A preliminary report* | 2002 | UK | General public | A, C |
| **Other** | | | | |
| Boyd, S., Dingle, R., and Campbell, R. *Taking a bite of the apple: The implementation of fruit in schools (healthy futures evaluation report to the Ministry of Health)* | 2007 | New Zealand | 8-10 | A, C, KA |
